# Genome engineering for estrogen receptor mutations reveals differential responses to anti-estrogens and new prognostic gene signatures for breast cancer

**DOI:** 10.1038/s41388-022-02483-8

**Published:** 2022-10-05

**Authors:** Alison Harrod, Chun-Fui Lai, Isabella Goldsbrough, Georgia M. Simmons, Natasha Oppermans, Daniela B. Santos, Balazs Győrffy, Rebecca C. Allsopp, Bradley J. Toghill, Kirsty Balachandran, Mandy Lawson, Christopher J. Morrow, Manasa Surakala, Larissa S. Carnevalli, Pei Zhang, David S. Guttery, Jacqueline A. Shaw, R. Charles Coombes, Lakjaya Buluwela, Simak Ali

**Affiliations:** 1 Department of Surgery & Cancer, Imperial College London, London, W12 0NN UK; 2 Semmelweis University Department of Bioinformatics, H-1094 Budapest, Hungary and TTK Cancer Biomarker Research Group, H-1117 Budapest, Hungary; 3 Leicester Cancer Research Centre, Department of Genetics and Genome Biology, University of Leicester, Robert Kilpatrick Clinical Sciences Building, Leicester Royal Infirmary, Leicester, LE2 7LX UK; 4 Early Oncology R&D, AstraZeneca, Biomedical Campus, 1 Francis Crick Ave, Cambridge, CB2 0AA UK; 5 Present Address: Institute of Cancer Research, Fulham Road, London, SW3 6JB UK

**Keywords:** Cancer, Molecular biology

## Abstract

Mutations in the estrogen receptor (ESR1) gene are common in ER-positive breast cancer patients who progress on endocrine therapies. Most mutations localise to just three residues at, or near, the C-terminal helix 12 of the hormone binding domain, at leucine-536, tyrosine-537 and aspartate-538. To investigate these mutations, we have used CRISPR-Cas9 mediated genome engineering to generate a comprehensive set of isogenic mutant breast cancer cell lines. Our results confirm that L536R, Y537C, Y537N, Y537S and D538G mutations confer estrogen-independent growth in breast cancer cells. Growth assays show mutation-specific reductions in sensitivities to drugs representing three classes of clinical anti-estrogens. These differential mutation- and drug-selectivity profiles have implications for treatment choices following clinical emergence of ER mutations. Our results further suggest that mutant expression levels may be determinants of the degree of resistance to some anti-estrogens. Differential gene expression analysis demonstrates up-regulation of estrogen-responsive genes, as expected, but also reveals that enrichment for interferon-regulated gene expression is a common feature of all mutations. Finally, a new gene signature developed from the gene expression profiles in ER mutant cells predicts clinical response in breast cancer patients with ER mutations.

## Introduction

**Fig. 1:**
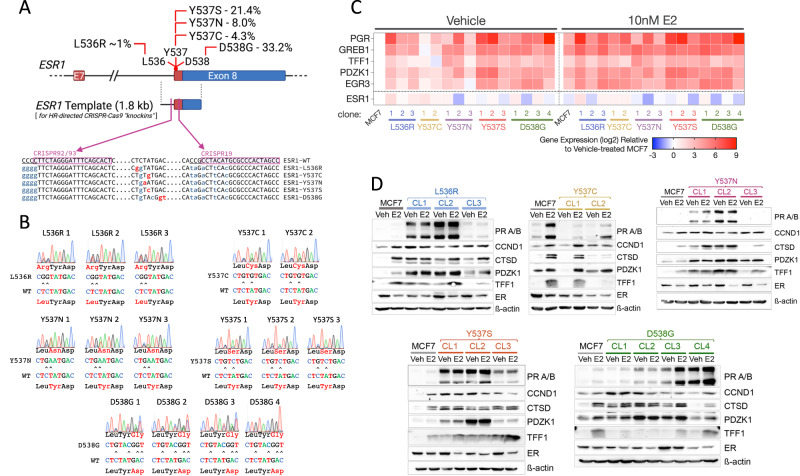
CRISPR-Cas9-directed generation of mutations in the ESR1 gene in MCF7 breast cancer cells. **A** The schematic outlines the strategy used to generate mutations in the endogenous ESR1 gene in MCF7 cells. The percentages refer to the prevalence of the different mutations in metastatic breast cancer patients, as described [42]. Shown are the positions of the CRISPR sequences, with PAM sequences underlined. Base changes introduced in the ESR1 template are shown in lower case, mutations in red being ones that change the amino acid and silent mutations in the CRISPR targeting/PAM sites shown in orange. **B** Shown are sequencing chromatograms for DNA prepared from each ESR1 mutant clone. **C** RNA was prepared from cells cultured in the absence of estrogen and treated with 10 nM estrogen for 16 h (*n* = 3). Gene expression was normalised to expression of the housekeeping gene TBP. The heat map shows the mean gene expression relative to expression in estrogen-free MCF7 cells. **D** Protein lysates were prepared as in (**C**). The immunoblotting panels show the results of one representative experiment. The same MCF7-WT lysates were used in each panel, for comparison across the different mutations (Supplementary Fig. 2 shows quantification of the 3 independent replicates.

More than 70% of breast cancers express estrogen receptor-α (ER) and ER-positive breast cancer is treated with endocrine therapies that fall into two main classes. The first are anti-estrogens, which compete with estrogen for binding to ER, but inhibit ER activity. The second approach is estrogen blockade, in which estrogen biosynthesis is inhibited to prevent ER activation, as exemplified by aromatase inhibitors (AI) [1, 2]. Although these therapies reduce breast cancer mortality, many patients ultimately relapse to metastatic endocrine-resistant disease [3, 4]. Genomic sequencing of metastatic breast cancers (MBC) has revealed that mutations in the ER gene (ESR1) are common in advanced endocrine-resistant breast cancer and are especially prevalent in AI-treated tumours [5–11]. These studies also reveal that ESR1 mutations are uncommon, or are limited to very few cells, in untreated primary tumours [12]. ESR1 mutations map almost exclusively to the ligand binding domain (LBD), with a hotspot of mutations proximal to, or within, the C-terminal-most α-helix, helix 12 [7–9, 13], encoded within exon 8 of the ESR1 gene (Fig. 1A). Conformational changes in the positioning of helix 12 are critical for ER activation upon estrogen binding, as well as for inhibition of ER activity by anti-estrogens [14, 15]. Bending of helix 12 over the LBD exposes consecutive hydrophobic residues in the loop between α-helices 11 and 12, which has been likened to a “spring-like strain” that is stabilised by ER agonists [16]. Mutation of leucine-536 (L536), tyrosine-537 (Y537) or aspartate-538 (D538) appears to relieve this tension by reducing hydrophobicity, promoting stronger hydrogen bonding, or lengthening of helix 12, which result in stabilisation of the unliganded receptor in the agonist conformation.

Prior to their discovery in MBC, in vitro studies, primarily using reporter gene assays, had shown that substitution of L536, Y537 and D538 by other amino acids can promote ligand-independent ER activity and reduced sensitivity to anti-estrogens [17–22]. Changes of Y537 to cysteine (Y537C), asparagine (Y537N) or serine (Y537S) and D538 to glycine (D538G), represent the most common mutations identified in patients and functional studies in breast cancer cell lines have confirmed earlier findings of estrogen-independent activity and reduced sensitivity to selective estrogen receptor modulators (SERMs), such as tamoxifen, as well as selective estrogen receptor downregulators (SERDs) like fulvestrant [7–9, 13, 23, 24]. Elevated expression of genes connected with metastasis, in cells expressing the Y537S and D538G mutations and increased metastatic potential in tumour xenografts [25–30], also implicates ESR1 mutations in driving transcriptomic reprogramming that promotes metastasis.

Methods for detection of circulating tumour cells (CTCs) and more readily, circulating tumour DNA (ctDNA) potentially allow for continual patient monitoring for emergence of ESR1 mutations and so, present opportunities for the early switchover of endocrine therapies once ESR1 mutations emerge [31, 32]. For implementation of therapy switching, it is necessary to understand the responsiveness of distinct ER mutations to different endocrine drugs. Estrogen independence, and hence resistance to AI, is clearly a feature of exon 8 encoded mutations. What remains less obvious is the extent of resistance of the different mutations to anti-estrogens. While the studies noted above provide evidence for reduced sensitivity to anti-estrogens, results of clinical studies remain ambiguous. For example, assessment of ctDNA from patients treated with fulvestrant following AI therapy did not show increases in ESR1 mutations during fulvestrant treatment [33], suggesting that ESR1 mutations are sensitive to fulvestrant. Indeed, in a further study, patients with detectable ESR1 mutations performed better on fulvestrant than on a steroidal AI, following progression on prior non-steroidal AI (SoFEA trial) [34]. The PALOMA-3 trial compared fulvestrant alone or with the CDK4/6 inhibitor Palbociclib. Here, retrospective analysis of ctDNA from patients on the PALOMA-3 trial showed that ESR1 mutations persist in both arms [35, 36]. Interestingly, while there was a significant increase in the Y537S mutation in patients who progressed on fulvestrant, other ESR1 mutations were not enriched in the PALOMA-3 patients [36], further clinical support for some ESR1 mutations in driving fulvestrant resistance. Adding to the complexity of current clinical understanding is the frequently observed polyclonality of ESR1 mutations (e.g. see [33]).

CRISPR-Cas9-mediated genome editing is a powerful approach for studying the impact of cancer mutations [37, 38]. Indeed, CRISPR-Cas9-directed MCF7 and T47D knock-in cell lines for Y537S and D538G have been reported [24, 25, 28, 39–41]. These studies have shown that the Y537S and D538G mutations confer ligand-independent proliferation and ER target gene expression, together with a reduction in sensitivity to anti-estrogens including tamoxifen, fulvestrant and AZD9496. Despite their frequent detection in MBC, functional analysis of the L536, Y537C and Y537N mutations has been more rudimentary, limited mainly to transient over-expression in ER+ (MCF7) and ER-negative cells [8, 23, 24]. To be able to assess the functions of the common ER mutations more comprehensively and to determine their activities and growth responses to anti-estrogens we have developed MCF7 CRISPR knock-in cell lines for the different Y537 mutations (Y537C, Y537N, Y537S), as well as for D538G and L536R, which together are present in 68% of patients with ESR1 mutations [42]. Our results show that all ER mutations promote estrogen-independent growth but reveal mutation related differences to anti-estrogens. Moreover, transcriptome analysis identifies a small number of genes whose expression is altered by each of the mutations. Importantly, this proto-signature can be refined to a simple, six-gene signature that predicts treatment response in breast cancer patients with ESR1 mutations. Hence, our work highlights the utility of a set of isogenic breast cancer cell lines for investigating new strategies for the treatment and diagnosis of ESR1 mutant breast cancer.

## Results

### Generation of MCF7 cells with genomically encoded L536R, Y537C, Y537N, Y537S and D538G mutations in the ESR1 gene

We previously reported the development and characterisation of one MCF7-Y537S knockin clone (hereafter referred to as Y537S CL3), using a CRISPR sgRNA (CRISPR19), located within exon 8 of the ESR1 gene [39]. Although we obtained many Y537S clones, all clones except Y537S CL3, featured indels in the other alleles (data not shown). As these indels may encode truncated ER proteins that would impact on the action of WT and mutant ER, we employed a revised strategy using sgRNAs targeting sequences within intron 7. Indels within intronic sequences upstream of the splice acceptor region should have less impact on gene expression. Using two overlapping sgRNA sequences upstream of the intron 7 splice acceptor region (Fig. 1A), we generated multiple isogenic MCF7 clonal lines for the L536R, Y537C, Y537N, Y537S and D538G mutations. Clones were identified by PCR of genomic DNA, using primers that specifically amplify mutant ESR1 sequences [39] and confirmation by RT-PCR for mutant expression (Supplementary Fig. 1A, B). Sanger sequencing of genomic DNA revealed that all clones are heterozygous for the mutation, except for D538G-CL4, in which the mutant allele predominated (Fig. 1B). None of the clones had apparent indels in exon 8 sequences. Two RT-PCR products (Supplementary Fig. 1B), consistent with the full length mRNA and a well-known alternatively spliced ER mRNA lacking exon 7 sequences [43], were present at similar ratios in MCF7 and the mutant clones, indicating that targeting to intron 7 did not affect appropriate splicing patterns in this part of the gene. In keeping with this, ER mRNA and protein levels were generally like those in MCF7-WT cells (Fig. 1C, D; Supplementary Fig. 2).

Progesterone receptor (PR) mRNA levels were consistently higher in all mutant clones than in MCF7 cells (Fig. 1C), as has been previously reported for Y537S and D538G mutations. The greatest increase in PR mRNA was seen for the Y537S (142-, 285- and 20-fold) and the heterozygous D538G clones (2.3–50-fold), with a remarkable 500-fold higher expression in D538G-CL4, the clone in which mutant ESR1 expression predominates. E2 stimulated PR expression in MCF7 cells (10.5-fold). In mutant clones, a substantial increase in PR expression by E2 addition was only seen in clones with the lowest PR expression in the absence of E2. Expression of the other estrogen-regulated genes followed the same trends as those for PR. Immunoblotting confirmed the remarkable increase in PR expression in most of the mutant clones (Fig. 1D, Supplementary Fig. 2). Levels of other ER target genes, CCND1, CTSD, PDZK1 and TFF1, were similarly elevated in the absence of E2. The main exception was the Y537C mutation, where there was little increase in ER target gene mRNA and protein levels in the absence of E2 and where E2 regulation was maintained.

### ESR1 mutations promote MCF7 growth in the absence of estrogen but reveal mutation-specific sensitivity to anti-estrogens

**Fig. 2:**
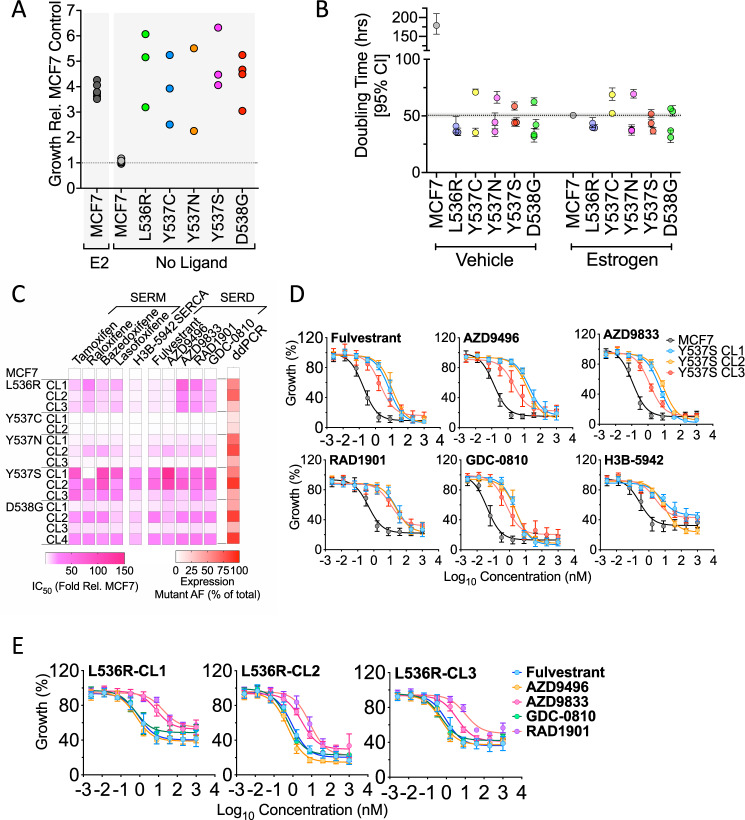
ESR1 mutant MCF7 clones grow in the absence of estrogen but show differential sensitivity to SERDs. **A, B** Cells cultured in estrogen-depleted medium were treated with 10 nM estrogen, or vehicle. Shown is the fold difference in growth of individual mutant clones, relative to the growth of MCF7 cells after 9 days. The results for 10 nM estrogen addition to MCF7 cells, are shown for comparison. **B** Doubling times for MCF7 and mutant clones, determined from growth experiment undertaken on an Incucyte Zoom ±10 nM E2 over a 9-day period (see Supplementary Fig. 3 for the full data). Doubling times were calculated using the exponential growth equation in Graphpad Prism. **C** The heatmap shows relative difference in IC_50_ values for mutant clones relative to the IC_50_ for MCF7 cells, determined for each mutant clone. Also shown are the mRNA expression levels of mutant ER, as a percentage of total ER in each clone, determined by ddPCR (see Supplementary Figs. 4, 5 for complete data). **D, E** Mean growth relative to vehicle (DMSO) is shown following treatment of MCF7, MCF7-Y537S and MCF7-L536R clones for 6 days, with increasing concentrations of anti-estrogens. The graphs plot the results of three independent experiments; error bars show standard deviations (see Supplementary Fig. 4 for actual IC_50_ values).

Growth of MCF7 cells was estrogen-dependent, with doubling times of 180 and 50 h in the absence and presence of E2, respectively (Fig. 2A, B; supplementary Fig. 2A, B). By contrast, all mutant clones proliferated in the absence of estrogen, with doubling times like those of estrogen treated MCF7 cells. This includes Y537C, in which expression of the classic ER target genes was not much enhanced in estrogen-free conditions (Fig. 1C, D). Interestingly, in DMEM containing 10% FCS, the mutant lines grew somewhat more slowly than MCF7-WT cells (Supplementary Fig. 3C, D), except the Y537S clones, which were indistinguishable from MCF7-WT. The slower growth of mutant clones, compared with MCF7-WT, was less marked in estrogen-free medium to which E2 was added than in DMEM containing 10% FCS. Pre-culturing MCF7 cells in estrogen-depleted culture medium causes growth arrest in MCF7-WT, so re-initiation of their growth is likely to lag behind growth of the estrogen-insensitive mutant clones. By contrast, MCF7-WT cells will not undergo growth arrest in the full medium conditions. The slower growth of the mutant clones in full medium is suggestive of a degree of reduced fitness imparted by ER mutations. PR activation has been shown to be anti-proliferative in ER + breast cancer cells [44]. Estrogen-depletion of FCS will remove progestins as well as estrogens, which will prevent PR activation, so masking the impact of PR over-expression in ER mutant cells. However, in culturing in normal FCS, progestins that are present may result in a greater anti-proliferative effect in ER mutant cells.

Although the ER mutations promoted estrogen-independent growth, all mutant clones were sensitive to anti-estrogens, including all SERMs and SERDs evaluated and the selective estrogen receptor covalent antagonist (SERCA), H3B-5942 [45]. However, the ESR1 mutant clones were less sensitive to anti-estrogens than MCF7 cells (Fig. 2C–E; Supplementary Figs. 4–5). The Y537S clones were generally the most resistant (Fig. 2D) and the Y537C and Y537N mutants were least resistant to the three classes of anti-estrogens.

Of note was the considerable variability in resistance seen in the different D538G clones. For instance, whereas D538G-CL2 and D538G-CL4 were, respectively, 35- and 25-fold less sensitive to fulvestrant, D538G-CL1 and D538G-CL3 were only 5.8- and 3.4-fold less sensitive. We determined if this differential anti-estrogen sensitivity might be related to different levels of mutant ESR1 expression. Droplet digital PCR (ddPCR) of cDNA prepared using RNA from each of the mutant clones, showed that the Y537C clones express low levels of mutant ER, comprising 12–18% of total ER (Supplementary Fig. 4). The low mutant expression levels in these clones may provide an explanation for the observed lack of resistance in the Y537C clones. However, low mutant expression did not necessarily explain the poor resistance of the Y537N clones, for example, comparing Y537N-CL1 (mutant allele frequency (MAF) = 67%) with Y537S-CL1 (MAF = 60%) or Y537S-CL3 (MAF = 41%). In the case of Y537N, substantial resistance to anti-estrogens was only evident in Y537N-CL2, where mutant ER predominates (MAF = 80.5%). This confirms the greater insensitivity to anti-estrogens for Y537S, at least when compared with its mutation to asparagine. Similarly, only D538G clones with predominant expression of the mutant when compared to wild-type ESR1 were substantially (>10-fold above MCF7) resistant (compare CL1 and CL3 with CL2 and CL4) to any of the anti-estrogens, again implicating expression levels of ER-D538G with response to anti-estrogens. Even for the Y537S clones, there was evidence for an association between responsiveness to anti-estrogens and the level of mutant expression, since Y537S-CL3, having the lowest mutant expression, was the clone that was least resistant to all SERDs, H3B-5942 and most of the SERMs. Despite these differences in the sensitivity of specific ER mutations, there was a clear, albeit moderate, association between levels of mutant expression and resistance to all SERDs (Supplementary Fig. 6).

Another notable feature of our analysis relates to the L536R mutation. Although L536R clones were less responsive to fulvestrant (~5-fold), the reduction in sensitivity was considerably lower than that for the Y537S mutation. This is consistent with the reported reduction in binding affinities of the Y537S and L536R mutants for fulvestrant, compared with WT-ER [16]. Interestingly, L536R was unique among the helix 11/12 mutations in displaying contrasting sensitivities to different SERDs. L536R mutant clones were just 3–5-fold less sensitive to fulvestrant or AZD9496 than MCF7-WT cells (Fig. 2E, Supplementary Fig. 6). By contrast, they were much less sensitive than MCF7-WT cells to camizestrant (AZD9833; 26–68-fold) and elacestrant (RAD1901; 18–38-fold). This suggests that care may need to be exercised when choosing the most appropriate SERD for patients relapsing on AI with evidence for mutation of L536.

### Global gene expression profiling of MCF7 cells expressing ESR1 mutants

**Fig. 3:**
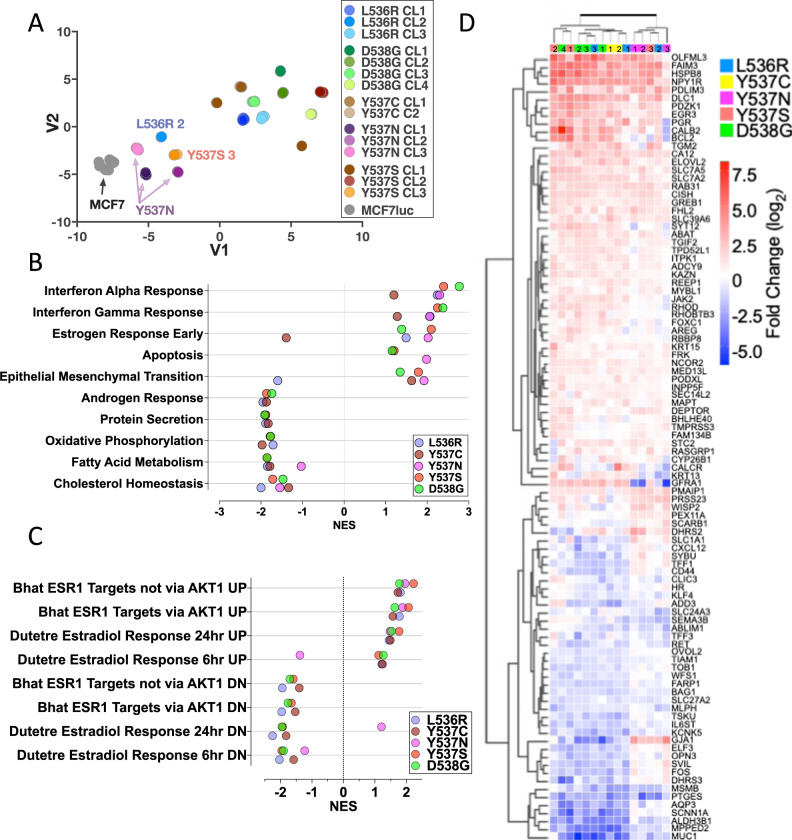
RNA-sequencing reveals gene expression patterns in ESR1 mutant cells. **A** Relationships between gene expression patterns in ESR1 mutant lines were visualised using a t-SNE plot. **B** Gene Set Enrichment Analysis (GSEA) was performed for each mutation. The top 10 enriched GSEA hallmark gene sets are shown. Filled circles represent the normalised enrichment score (NES) for each mutation (FDR *q*-value <0.25). **C** Shown is GSEA analysis undertaken using the C2 curated MSigBD for gene sets that are involved in estrogen signalling, excluding gene sets containing less than 80 genes (FDR q-value <0.25). **D** Heatmap shows genes within the hallmark gene sets “estrogen response early” and “estrogen response late”, which are differentially regulated in all mutations compared to MCF7, with the following cut-off: −0.5 > log2FC > 0.5. The individual clones for each ESR1 mutation are denoted with the coloured, numbered boxes at the top of the heatmap.

We previously used RNA-seq and ChIP-seq, to compare chromatin recruitment of ER and gene expression in MCF7-WT and Y537S-CL3, in the presence or absence of E2 [39]. Estrogen-independent ER recruitment to DNA, accompanied by the expression of ER target genes, was observed in the mutant cells and the expression of many ER target genes was elevated to levels considerably higher than those obtained with estrogen treatment of WT cells, as confirmed in other studies [24, 25, 28, 29, 39–41]. However, gene expression profiling in most of these studies was performed after culturing the cells in estrogen-depleted medium (which prevents the growth of WT ER cells but allows continued growth of the mutant cells), followed by short-term addition of E2. This may explain the preponderance of cell cycle-associated pathways enriched in mutant cells observed in many studies [29, 39, 46]. To identify genes in mutant cells where expression may not be due to differences arising from short term exposure to estrogen, we carried out RNA-seq using cells that had been maintained in estrogen-containing medium. We undertook RNA-seq using six biological replicates, as this is consistent with described recommendations for a false discovery rate of ≤5% for 0.5-fold difference in expression using DESeq2 [47]. Because of sequencing of the large number of biological replicates of MCF7-WT and the 15 mutant clones, we used the t-distributed stochastic neighbour embedding (t-SNE) method for visualising relationships between the clones. Biological replicates for all cell lines were very tightly clustered and all mutant lines separated away from MCF7-WT (Fig. 3A).

DESeq2 identified several hundred genes that differentiated each mutant clone from MCF7-WT (log2FC > 1, padj<0.05; Supplementary Fig. 7A). Similar numbers of genes were down-regulated as were up-regulated within each mutant clone. The largest numbers of differential genes were noted for Y537S-CL1 and -CL2; the Y537N clones featured the fewest gene expression changes. Estrogen, interferon response and epithelial mesenchymal transition (EMT) were the most positive enriched Hallmarks pathways (Fig. 3B; Supplementary Tables S1–S6). The main point of interest about the estrogen response pathway was its downregulation in Y537C clones. However, re-analysis with the “C2 curated gene sets: CGP (Chemical and genetic perturbations)” MSigDB gene-set collection showed that all mutants, including Y537C, were positively and negatively enriched for estrogen-stimulated and estrogen-repressed gene sets, respectively (FDR *q*-value <0.25; Fig. 3C, Supplementary Fig. 7B). Indeed, expression of classic ER target genes was increased in Y537 C clones (Supplementary Fig. 7C), as was the case for the other mutations, demonstrating that increased expression of ER target genes is common to all ESR1 mutants (Fig. 3D). Among the down-regulated Hallmarks pathways are metabolic pathways, including cholesterol homeostasis, fatty acid metabolism and oxidative phosphorylation. Notably, also included among the down-regulated pathways is “androgen response”, indicating that ESR1 mutations reduce androgen receptor (AR) activity (Supplementary Fig. 8A–C).

**Fig. 4:**
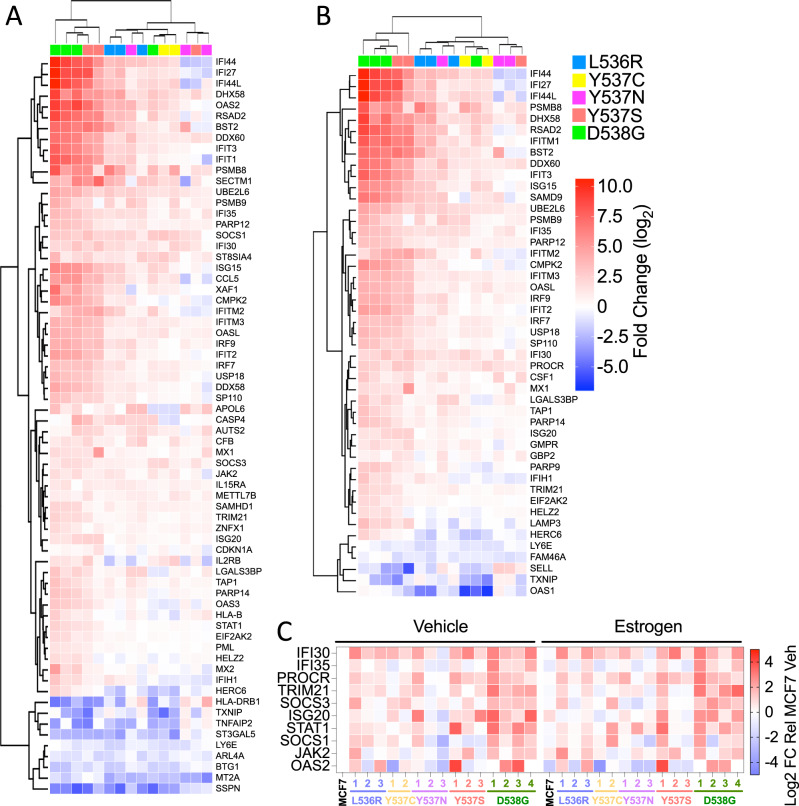
Genes associated with interferon response pathways are enriched in ESR1 mutant cells. **A, B** The heatmaps show genes within the hallmark gene sets “interferon alpha response” and “interferon gamma response”, which are differentially regulated in all mutations compared to MCF7 (cut-off: −0.5 > log2FC > 0.5). **C** RT-qPCR was performed using RNAs described in Fig. 1C. The heat map shows mean expression relative to expression in estrogen-free MCF7 cells (the data summarised in the heat map are plotted in Supplementary Fig. 9).

Interestingly, interferon-alpha and interferon-gamma response were identified as the most highly enriched Hallmark pathways (Fig. 3B). Forty-seven and sixty-eight genes in the interferon-alpha and -gamma pathways, respectively, were differentially regulated in ESR1 mutant clones, when compared with expression in MCF7-WT cells (Fig. 4). For the most part, expression of interferon-stimulated genes (ISG) was increased in the mutant clones. RT-qPCR confirmed upregulation of ISG and showed that expression of these genes was, on the whole, not stimulated by estrogen (Fig. 4C, Supplementary Fig. 9A). Indeed, expression of some ISGs was repressed by estrogen in MCF7-WT cells. The increased ISG expression was confirmed by immunoblotting for STAT1 (Supplementary Fig. 9B). Elevated ISG expression has been reported for Y537S and D538G mutations [30, 48]. Our results confirm these findings and show that other ESR1 mutations similarly upregulate ISG expression in breast cancer cells. MCF7-D538G clone 1 showed the highest STAT1 upregulation, yet growth regulation by the JAK1/2 inhibitors ruxolitinib and baricitinib was comparable to the growth inhibition observed for MCF7 cells (Supplementary Fig. 9C), indicating that the STAT1 up-regulation is not implicated in growth regulation of ESR1 mutant MCF7 cells.

### Identification of a gene signature of mutant ER activity that is prognostic in ER+ breast cancer

DESeq2 analysis was employed for identifying genes that are differentially regulated across all ESR1 mutations, comparing to expression in MCF7-WT. For this purpose, read numbers from individual clones for each mutation were used as replicates. A total of 172 and 45 genes were subsequently seen to be up- and down-regulated (−1 > log_2_FC > 1; padj<0.05) in all ESR1 mutations relative to WT MCF7 (Supplementary Fig. 10A–C; Supplementary Table S7). Plotting the mean normalised read counts for genes in each category confirmed increased expression of all UP and reduced expression of all DOWN genes, in the ESR1 mutant clones (Supplementary Fig. 10DA, E; Supplementary Table S8). GSEA “compute overlap” analysis for significantly enriched (FDR < 0.05) Hallmarks gene sets, identified gene sets already observed in the analysis of the individual mutants (Fig. 3B), including estrogen and interferon response pathways for the up-regulated and metabolic pathways for the down-regulated gene sets.

The presence of ESR1 mutations has been associated with poor survival in breast cancer patients (for review see [49]). We evaluated the possibility that the upregulated genes common to the exon 8 ESR1 mutations would be associated with patient response. The 172 genes were filtered to exclude those genes that only have very low expression in most of the mutant clones, as well as 7 non-coding genes, yielding a set of 136 genes (Supplementary Table S9). This filtering meant that only genes with higher than 50 reads in at least 42 biological replicates (equivalent to 7 mutant clones, almost half of the total number of clones) were included. The best cut-off for differentiating high and low expression cohorts were determined as described previously [50]. Interestingly, high expression of the mutant up-regulated genes was associated with improved patient outcome in ER+ breast cancer patients, as seen in the Affymetrix patient data series, TCGA and METABRIC (Supplementary Fig. 11A–C). By contrast, there was no association of the down-regulated genes with relapse-free survival (RFS) in Affymetrix or TCGA patient series. By contrast, low expression of these genes was significantly associated with worse overall survival in the METABRIC series (HR = 0.83 (0.71–0.96), *p* = 0.011; Supplementary Fig. 11D–F).

**Fig. 5:**
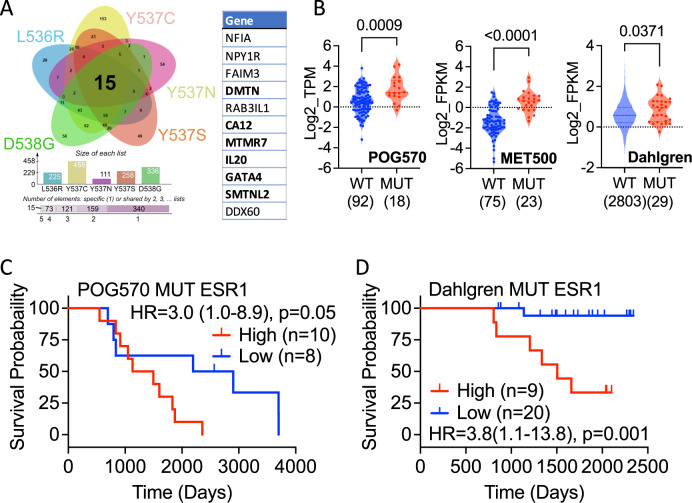
Identification of differential genes for all ESR1 mutants. **A** DEGs that show increased (log2FC > 1 and padj <0.05) expression in every clone for each ESR1 mutation were overlapped in the Venn diagram. The 11 genes used in signature development are listed. The 6 genes developed from analysis of the POG570 mutant ESR1 cancers are highlighted in bold. **B** Expression of the 6-gene list is elevated in ESR1 mutant metastatic (POG570, MET500) and primary (Dahlgren) breast cancer. The number of ER + patients with WT and mutant ESR1 are shown in parentheses. The Mann-Whitney test was used for significance determination. **C, D** Kaplan–Meier plots of survival for ER + breast cancer patients with ESR1 mutations. The hazard ratios (HR), together with 95% confidence intervals (in brackets) and *p* value from the log rank test is shown in each panel; ‘n’ is the number of samples in each group.

Further analysis was carried out to refine this initial, mutant up-regulated gene set by identifying genes that were upregulated at an individual clone level for each of the ESR1 mutations and resulted in a proto gene signature of just 15 genes whose expression was consistently higher (log2 FC > 1; padj<0.05) than that in MCF7 WT cells (Fig. 5A; Supplementary Table S10). Analysing breast cancer gene expression data sets provided little evidence for expression of the non-coding RNAs AC005256.1 and AC011747.4, or the RPL13A pseudogene ENSG00000226945, so these were omitted from further consideration. SMOC2 was also excluded due to very low/absent expression in the mutant clones. Survival analysis revealed that high expression of the remaining 11-gene signature is associated with better patient outcomes in TCGA and METABRIC (Supplementary Fig. S12A). A similar survival advantage was observed in the SCAN-B (Dahlgren) breast cancer series that includes RNA-seq analysis for 2720 primary, pre-treatment ER+ patients [51].

We next sought to establish if the genes upregulated in our ESR1 mutant MCF7 cells are also more highly expressed in breast cancers with ESR1 mutations. For this, we utilised the largest available MBC cohorts with ESR1 mutational status and gene expression profiling, MET500 and POG570 [52, 53]. Expression of these 11 genes was significantly higher in mutant ESR1 samples in both patient cohorts (Supplementary Fig. 12B). The Dahlgren series of primary, pre-treatment ER+ breast cancer includes 29 cases with ESR1 mutations [51]. Again, the 11-gene signature was more highly expressed in primary breast cancer cases with mutant ESR1.

Univariate Cox proportional hazards analysis for each of these 11 genes using the POG570 metastatic breast cancer dataset identified six genes (MTMR7, SMTNL2, GATA4, IL20, CA12, DMTN) that had a positive HR value (exp(coef)>1) in the ESR1 mutant samples. High expression of these genes was associated with better survival in POG570 metastatic and Dahlgren WT ESR1 primary breast cancer patients (Supplementary Fig. 12C, D). By contrast, the same genes combined for worse overall survival in the POG570 mutant ESR1 patients (Fig. 5C). Moreover, high expression of the signature was strongly associated with worse survival in the Dahlgren ESR1 mutant primary breast cancer patients (Fig. 5D).

## Discussion

The acquisition of ESR1 mutations in breast cancer is clearly key to the development of resistance to AI and may provide a mechanism for reduced sensitivity to anti-estrogens, including SERDs. Constitutive ER activity due to these mutations is likely to result in altered gene expression patterns in breast cancer cells. Here, we report the largest isogenic series of ESR1 mutant lines described to date, which collectively provide a new resource for investigating the sensitivities of different mutations to the diverse ER targeting therapies, including SERDs, SERCAs and ER PROTACs. Our studies demonstrate differential responses of the different ESR1 mutations to SERMs, SERDs, as well as the SERCA H3B-5942. In agreement with previous reports, the Y537S mutation also showed the greatest resistance to all SERDs, SERMs and H3B-5942. This agrees with structural studies which reveal that the Y537S mutation has the most stable active conformational change, due to a novel hydrogen bond, and that it also has the highest estrogen-independent activity, co-activator binding and resistance to anti-estrogens, when compared to other ESR1 mutations [16, 54–56]. In general, the Y537S and D538G mutations are the most resistant to anti-estrogens. Most interesting was the differential response of L536 mutants to different SERDs, with a very small reduction in sensitivity to fulvestrant or AZD9496, but considerably greater resistance to AZD9833, GDC-0810 and RAD1901. While the reasons for these differences require further investigation, our results suggest that the nature of the mutated residue should be considered in selecting a SERD for patient treatment. Of course, this will be tempered by the effective dose achievable in patients. Indeed, RAD1901 (elacestrant) demonstrated prolongation of progression-free survival in a phase III setting compared to standard-of-care endocrine treatments in advanced pre-treated ER+/HER2- patients with ctDNA positivity for ESR1 mutations [57]. Clinical benefit is also indicated by the reduction in ESR1 mutations in longitudinal ctDNA analysis for patients on treatment with AZD9833 [58]. It will be interesting to know whether there is any association between specific ESR1 mutations and response in these trials.

Also revealed by our studies was the potential impact of mutant ESR1 expression levels on response to endocrine therapies. Growth in the absence of estrogen was universally observed for all mutant clones, irrespective of mutant expression levels. Although most mutant clones were less sensitive to anti-estrogens, reduction in sensitivities was greater in clones with higher mutant expression, compared with WT ER levels. This is most clearly seen in the case of the D538G mutation. Clones with mostly mutant D538G expression were 3–5-fold less sensitive to SERMs and SERDs, than clones in which WT ER predominated. This differential resistance to anti-estrogens was also indicated for the Y537S mutation, since SERM, SERD and SERCA insensitivity was weakest in the case of Y537S-CL3, which has the lowest mutant:WT ER ratio. These results echo estrogen-regulated reporter gene assays in which reduced responsiveness to fulvestrant, AZD9496 and lasofoxifene was observed only when mutant expression greatly exceeded that of WT ER [59]. Indeed, our results show correlations between ratios of mutant:WT mRNA expression and growth response to SERDs. These findings raise the possibility that levels of mutant expression may increase following patient progression on SERDs, something that simple assessment of genomic ESR1 mutant status will not reveal.

Increased expression of estrogen-regulated genes in estrogen-free conditions is expected for the ESR1 mutations, as confirmed by RT-qPCR and immunoblotting. However, RNA-seq performed in estrogen-containing culture medium also showed enrichment in estrogen response pathways, indicative of increased ER activity due to mutant expression. Interferon signalling pathways were also universally enriched, including STAT1. Indeed, STAT1 protein and phosphorylation levels were increased in mutant clones, but we did not find evidence for altered growth following treatment with JAK/STAT inhibitors. However, a recent study revealed enrichment of interferon pathways in ESR1 mutant cells and linked elevated T helper and T regulatory cells and enhanced macrophage infiltration in metastatic samples with mutant, compared with those with WT ER [48]. Their observation of higher levels of PD-L1 positive macrophages in lesions from patients with mutant ER metastatic lesions compared to those with WT ER, led to the proposal of greater susceptibility of ER mutant metastatic breast cancer to immunotherapies. Enrichment in interferon signalling pathways in our models is certainly in agreement with this previous report.

Among the most negatively enriched pathways was androgen signalling, which would be consistent with the reported estrogen-mediated reductions in AR levels in breast cancer cells [60, 61]. Inhibition of AR signalling could be involved in the described pro-metastatic activity of ESR1 mutants and is concordant with the tumour suppressive effects of AR in ER+ breast cancer comprehensively demonstrated recently [62]. However, one study found elevated AR expression in ESR1 mutant MCF7 and T47D cells, as well as inhibition of anchorage-independent growth of mutant ER and WT ER MCF7 cells by the anti-androgen enzalutamide, which is consistent with a pro-metastatic role for AR [48]. By contrast, the AR agonist dihydrotestosterone has been reported to inhibits metastasis of ER-Y537S or ER-D538G breast cancer cells in vivo [30]. Reduction in AR signalling identified in our study would be consistent with the latter study, but further investigation of AR action in ESR1 mutant breast cancer is merited.

Averaging gene expression in the independent clones for each ER mutant identified 143 gene upregulated genes, high expression of which was associated with better patient response in ER+ breast cancer patients, likely reflecting the enrichment in estrogen response pathways. Just 15 genes were identified when only genes that were upregulated in every mutant clone were considered, which resolved down to a six gene set, which was more highly expressed in mutant than in WT ER metastatic breast cancer from the POG570 and MET500 cohorts. In POG570, where patient survival data are available, high expression of these genes was associated with better survival in patients with WT ER, as observed for primary breast cancer. However, the same genes were associated with poor survival in mutant ER patients in this cohort. In the Dahlgren SCAN-B ER+ primary breast cancer patients treated with ET consisting of either AI or tamoxifen, presence of pre-existing ESR1 mutations was associated with poor survival (*p* = 0.008). High expression of our six gene signature was also associated with worse overall survival (*p* = 0.0012). Indeed, there was just one death in 20 patients for the low expression group, compared with 6/9 patients in the high expression group. Our results raise the intriguing possibility that despite the presence of mutant ER, patients with low expression of our gene signature may benefit from AI. High signature expression would indicate the need for alternatives to AI, such as SERDs.

In conclusion, we have generated a large series of ESR1 mutant MCF7 cells, which will be a useful resource for investigating functional similarities and differences between distinct ER mutations and aid therapeutic strategies. Our analysis identifies mutational differences in sensitivity to anti-estrogens and highlights the importance of mutant expression levels on response to anti-estrogens. Finally, we have identified a simple six-gene-based expression signature that may have utility in identifying those patients who may benefit from AI, despite pre-existing ESR1 mutations. This is potentially important, as gene expression signatures, such as the Oncotype Dx Breast Recurrence Score and Mammaprint, are already in use for guiding treatments for breast cancer patients [63].

## Materials and methods

### Tissue culture and growth assays

MCF7-Luc cells (hereafter referred to as MCF7; Cambridge Bioscience, Cambridge, UK) and derived mutant ESR1 clones were authenticated by short tandem repeat profiling using the AmpFlSTR Identifiler Plus kit (Applied Biosystems, Warrington, UK), as described [64]. Mycoplasma negativity was maintained by regular testing using the MycoAlert Mycoplasma Detection Kit (Lonza, UK). Cell lines were routinely cultured in Dulbecco’s Modified Eagle’s medium (DMEM) containing 10% fetal calf serum (FCS) and penicillin-streptomycin-L-glutamine (PSG). For estrogen depletion, the cells were transferred to DMEM lacking phenol red and containing 5% dextran-coated charcoal-stripped FCS (DSS) for 72 h. Stock solutions of 17ß-estradiol (E2) and anti-estrogens, prepared in DMSO, were added to the culture medium at a dilution of 1 in 1000. An equal volume of DMSO was added to the vehicle controls. Compound details are listed in supplementary information. Cell growth was measured using the sulphorhodamine B (SRB) assay, as described previously [39], or live cell imaging using the IncuCyte ZOOM (Essen Bioscience, Welwyn Garden City, UK). For the latter, three images per well were acquired every 12 h for a period of 6–9 days, and confluency (%) calculated using the IncuCyte ZOOM software package (Essen Bioscience). For determination of the half-maximal effective concentration (IC_50_) values, cells were seeded in 96-well culture plates and treated with increasing concentrations of anti-estrogens for 6 days. Cell growth was determined using the SRB assay. IC_50_ values were calculated from non-linear regression curve fitting using GraphPad Prism v9. Doubling times were calculated in Prism, using the exponential growth equation.

### Generation of ER-mutant MCF7 cell lines using CRISPR-Cas9 genome editing

The MCF7-Y537S A4 clone (here referred to as Y537S CL3) has been reported [39]. The other ESR1 mutant lines were generated using the same approach, following site-directed mutagenesis of an 1803 bp fragment of the ESR1 gene flanking the exon 8 coding region, except that MCF7 cells were transfected with the hCas9 and donor template plasmids, together with the CRISPR sgRNA CRISPR4834192 or CRISPR4834193. These CRISPRs, targeting intron 7 of ESR1, were designed using web-based software (https://zlab.bio/guide-design-resources). Single colony cloning and screening of genomic DNA using mutant-specific PCR was undertaken, again as detailed [39]. PCR of genomic DNA followed by Sanger sequencing was used to confirm correct integration of the appropriate mutation in the ESR1 gene locus.

### Real-time RT-PCR (RT-qPCR)

Gene expression analysis was carried out using Powerup^TM^ SYBR Green PCR master mixes (Applied Biosystems). Expression of the gene of interest was normalised to TBP expression, using the 2^-∆∆CT^ method. Primer sequences are listed in Supplementary Information.

### Immunoblotting

Whole cell lysates were prepared in RIPA buffer (Sigma-Aldrich), supplemented with protease and phosphatase inhibitor cocktails (Roche, West Sussex, UK), as previously described [39]. Antibodies are detailed in Supplementary Information. Immunoblotting was performed for three independent biological replicates. Band intensities were quantified by densitometry using ImageJ [65]. Antibody details are provided in Supplementary Information.

### Droplet digital PCR (ddPCR)

The proportion of WT and mutant ER expression was assessed by ddPCR of cDNA for each clone, using a Bio-Rad QX200 droplet digital PCR system as described previously [66]. Assays were designed using OligoArchitect and were performed at 62 °C using 20 ng gDNA, a matched amount of cDNA and a no template control (NTC) reaction were also undertaken. Primer sequences are given in Supplementary Information.

### RNA-sequencing (RNA-seq)

Total RNA was extracted for six biological replicates, using the QIAGEN RNeasy Mini Preparation Kit (QIAGEN Ltd, Crawley, UK). Library preparation using the NEBNext® Ultra™ RNA Library Prep Kit and 150 bp paired-end sequencing was carried out on the Illumina Novaseq 6000 platform at the Beijing Genomics Institute. Using a Bcbio 1.1.1 RNA-seq pipeline, reads were aligned using Hisat2 2.1.0 [67], counted using DEXSeq [68] and Salmon 0.11.3 [69] and normalised using the R package DESeq2 [70]. DESeq2 was also used to determine differentially expressed genes between WT and mutant cell lines using shrunken log2 fold changes. Heatmaps were generated using the gplots R package. GSEA analysis was performed using the Molecular Signatures Database ‘Hallmarks’ gene set collection [71]. Venn diagrams were created using jvenn [72]. Grouping of knock-in clones was visualised using the t-Distributed Stochastic Neighbour Embedding (t-SNE) method for dimensionality reduction, using regularised log2-transformed (rlog) read counts as input. The t-SNE model was run using the ‘Rtsne’ R package, with a perplexity of 30, theta of 0.5, and other parameters kept at default settings. RNA-seq data have been deposited with the NCBI Gene Expression Omnibus (GEO) (http://ncbi.nlm.nih.gov/geo/) under accession number GSE147745.

### Gene signature identification and survival analyses

Kaplan-Meier (KM) plots of overall survival were determined using the online tool in http://kmplot.com/analysis [50] by analysing gene expression of curated GEO datasets (Affymetrix only), comprising a series of 1401 ER+ patients. ‘The Cancer Genome Atlas’ (TCGA), and Molecular Taxonomy of Breast Cancer International Consortium (METABRIC) datasets [73, 74] were also analysed using KMplot. The 6-gene signature was chosen based on univariate cox proportional hazards analysis of the POG570 [52] mutant ESR1 breast cancer samples, taking all genes, which were up-regulated in all clones for each ESR1 mutation, with a hazard ratio (HR) > 1. Survival analyses using univariate Cox proportional hazards model with these 6 genes were undertaken and KM plots generated with the ‘survminer’ and ‘survival’ R packages, using the optimal expression cut-off functionality. This was carried out on the following datasets, which were filtered for ER+ breast cancer patients: POG570 overall survival (OS), SCAN-B OS [51], TCGA pan cancer atlas OS and progression-free survival (PFS; downloaded from cbioportal, 05/01/22), METABRIC OS and regression-free survival (RFS; downloaded from cbioportal, 20/07/21). Differences were tested for significance using the log-rank test. Hazard ratios and 95% confidence intervals were calculated. *P* value <0.05 was indicative of statistical significance. Expression of the 6 genes in ESR1 mutant vs WT patients was determined in the POG570 (log2(TPM + 1)), SCAN-B (log2 FPKM) and MET500 (log2 FPKM [53]) datasets where ESR1 mutation status was available. ER status was not available for MET500 patients, so FPKM > 1 cut-off was used to classify patients as ER+.

### Statistical analyses

All data are presented as mean, errors as standard error of the mean, unless stated otherwise. Cell growth was determined by IncuCyte Zoom as percentage confluency and the data points input into GraphPad Prism v9 to calculate doubling time using exponential growth equation. Errors are represented as 95% confidence intervals. SRB growth assay was used to determine the response to anti-estrogens, IC_50_ values were calculated by non-linear regression curve fitting in Prism.

## Supplementary information

Supplementary Tables

Supplementary Figures

Supplementary Information
