# Supplementary material for: Genome engineering for estrogen receptor mutations reveals differential responses to anti-estrogens and new prognostic gene signatures for breast cancer
Source: Oncogene. 2022 Oct 5;41(44):4905–15. doi: 10.1038/s41388-022-02483-8 (PMC7613769; doi:10.1038/s41388-022-02483-8)
Supplement: Supplementary file 1 — Supplementary Tables [file 41388_2022_2483_MOESM1_ESM.pdf]

Supplementary Table S1 Gene Set Enrichment Analysis (GSEA)
All Mutants v MCF7-WT
Pathways UP

NAME	SIZE	ES	**NES**	NOM p-val	**FDR q-val**	FWER p-val	RANK AT MAX
**HALLMARK_INTERFERON_ALPHA_RESPONSE**	82	0.6	**2.3**	0	**0**	0	2787
**HALLMARK_INTERFERON_GAMMA_RESPONSE**	141	0.5	**2.1**	0	**0**	0	2840
**HALLMARK_ESTROGEN_RESPONSE_EARLY**	198	0.4	**1.8**	0	**0.004**	0.018	1905
**HALLMARK_EPITHELIAL_MESENCHYMAL_TRANSITION**	119	0.4	**1.6**	0	**0.031**	0.171	2255
**HALLMARK_HEDGEHOG_SIGNALING**	26	0.5	**1.5**	0.058	**0.071**	0.424	1985
**HALLMARK_P53_PATHWAY**	184	0.3	**1.4**	0.014	**0.088**	0.549	1574
**HALLMARK_APICAL_JUNCTION**	133	0.3	**1.4**	0.027	**0.085**	0.59	2532
**HALLMARK_ESTROGEN_RESPONSE_LATE**	193	0.3	**1.4**	0.024	**0.122**	0.771	1388
**HALLMARK_APOPTOSIS**	123	0.3	**1.3**	0.042	**0.111**	0.778	2713
**HALLMARK_ALLOGRAFT_REJECTION**	85	0.3	**1.3**	0.063	**0.128**	0.852	2573
**HALLMARK_TNFA_SIGNALING_VIA_NFKB**	156	0.3	**1.3**	0.058	**0.152**	0.924	2574
**HALLMARK_WNT_BETA_CATENIN_SIGNALING**	33	0.4	**1.2**	0.169	**0.190**	0.965	1518
**HALLMARK_COAGULATION**	64	0.3	**1.2**	0.145	**0.179**	0.965	1987
**HALLMARK_TGF_BETA_SIGNALING**	51	0.3	**1.2**	0.200	**0.247**	0.994	1696
HALLMARK_ANGIOGENESIS	23	0.4	1.1	0.348	0.409	1	1432
HALLMARK_INFLAMMATORY_RESPONSE	96	0.3	1.1	0.316	0.429	1	2769
HALLMARK_NOTCH_SIGNALING	27	0.3	1.1	0.360	0.422	1	1518
HALLMARK_IL6_JAK_STAT3_SIGNALING	46	0.3	1.0	0.401	0.438	1	2770
HALLMARK_APICAL_SURFACE	26	0.3	0.9	0.596	0.740	1	2769
HALLMARK_MITOTIC_SPINDLE	195	0.2	0.9	0.753	0.719	1	2933
HALLMARK_REACTIVE_OXYGEN_SPECIES_PATHWAY	45	0.2	0.8	0.734	0.807	1	3352

Pathways DOWN

NAME	SIZE	ES	**NES**	NOM p-val	**FDR q-val**	FWER p-val	RANK AT MAX
**HALLMARK_PROTEIN_SECRETION**	93	-0.5	**-2.0**	0	**0.002**	0.001	3200
**HALLMARK_CHOLESTEROL_HOMEOSTASIS**	65	-0.5	**-1.9**	0	**0.002**	0.002	1719
**HALLMARK_OXIDATIVE_PHOSPHORYLATION**	197	-0.4	**-1.9**	0	**0.002**	0.003	3763
**HALLMARK_FATTY_ACID_METABOLISM**	129	-0.4	**-1.8**	0	**0.005**	0.013	1898
**HALLMARK_XENOBIOTIC_METABOLISM**	133	-0.4	**-1.7**	0	**0.013**	0.041	1089
**HALLMARK_ANDROGEN_RESPONSE**	92	-0.4	**-1.6**	0	**0.027**	0.103	1741
**HALLMARK_KRAS_SIGNALING_UP**	101	-0.4	**-1.6**	0.003	**0.025**	0.11	871
**HALLMARK_HYPOXIA**	163	-0.3	**-1.6**	0	**0.023**	0.114	1411
**HALLMARK_MTORC1_SIGNALING**	192	-0.3	**-1.5**	0	**0.031**	0.167	2537
**HALLMARK_ADIPOGENESIS**	172	-0.3	**-1.5**	0	**0.033**	0.194	1828
**HALLMARK_HEME_METABOLISM**	153	-0.3	**-1.4**	0.009	**0.051**	0.302	1899
**HALLMARK_PEROXISOME**	86	-0.3	**-1.4**	0.019	**0.056**	0.351	2295
**HALLMARK_GLYCOLYSIS**	178	-0.3	**-1.3**	0.012	**0.113**	0.614	2697
**HALLMARK_MYC_TARGETS_V1**	200	-0.3	**-1.3**	0.013	**0.148**	0.739	3635
**HALLMARK_BILE_ACID_METABOLISM**	67	-0.3	**-1.3**	0.110	**0.162**	0.799	2085
**HALLMARK_MYOGENESIS**	113	-0.3	**-1.2**	0.085	**0.183**	0.862	340
**HALLMARK_IL2_STAT5_SIGNALING**	133	-0.3	**-1.2**	0.089	**0.186**	0.881	990
**HALLMARK_COMPLEMENT**	119	-0.3	**-1.2**	0.117	**0.189**	0.901	767
**HALLMARK_PI3K_AKT_MTOR_SIGNALING**	88	-0.3	**-1.2**	0.143	**0.191**	0.919	1701
HALLMARK_KRAS_SIGNALING_DN	76	-0.2	-1.0	0.372	0.531	0.998	1139
HALLMARK_UV_RESPONSE_UP	136	-0.2	-1.0	0.473	0.598	1	2084
HALLMARK_UNFOLDED_PROTEIN_RESPONSE	110	-0.2	-1.0	0.478	0.579	1	3433
HALLMARK_UV_RESPONSE_DN	124	-0.2	-1.0	0.491	0.590	1	1637
HALLMARK_G2M_CHECKPOINT	198	-0.2	-0.9	0.705	0.762	1	2806
HALLMARK_E2F_TARGETS	199	-0.2	-0.9	0.728	0.750	1	2800
HALLMARK_DNA_REPAIR	144	-0.2	-0.8	0.884	0.910	1	1491
HALLMARK_SPERMATOGENESIS	62	-0.2	-0.8	0.822	0.894	1	2444
HALLMARK_MYC_TARGETS_V2	58	-0.2	-0.7	0.958	0.972	1	2664

Supplementary Table S2 Gene Set Enrichment Analysis (GSEA)
L536R Clones 1-3 v MCF7-WT
Pathways UP

NAME	SIZE	ES	**NES**	NOM p-val	**FDR q-val**	FWER p-val	RANK AT MAX
**HALLMARK_INTERFERON_ALPHA_RESPONSE**	82	0.6	**2.2**	0	**0**	0	1830
**HALLMARK_INTERFERON_GAMMA_RESPONSE**	141	0.5	**2.1**	0	**0**	0	1806
**HALLMARK_ESTROGEN_RESPONSE_EARLY**	198	0.3	**1.5**	0	**0.041**	0.23	1720
HALLMARK_APOPTOSIS	123	0.3	1.1	0.213	0.573	0.995	2123
HALLMARK_WNT_BETA_CATENIN_SIGNALING	33	0.3	1.1	0.355	0.638	0.998	1837
HALLMARK_MITOTIC_SPINDLE	195	0.2	0.9	0.698	1.000	1	3021
HALLMARK_REACTIVE_OXYGEN_SPECIES_PATHWAY	45	0.2	0.8	0.879	1.000	1	3388
HALLMARK_MYC_TARGETS_V2	58	0.2	0.7	0.973	1.000	1	3614
HALLMARK_DNA_REPAIR	144	0.1	0.6	1.000	1.000	1	3737
HALLMARK_E2F_TARGETS	199	0.1	0.5	1.000	0.998	1	3832

Pathways DOWN

NAME	SIZE	ES	**NES**	NOM p-val	**FDR q-val**	FWER p-val	RANK AT MAX
**HALLMARK_HYPOXIA**	163	-0.5	**-2.1**	0	**0.003**	0.002	1728
**HALLMARK_KRAS_SIGNALING_UP**	101	-0.5	**-2.1**	0	**0.001**	0.002	1480
**HALLMARK_CHOLESTEROL_HOMEOSTASIS**	65	-0.5	**-2.0**	0	**9.19E-04**	0.002	1890
**HALLMARK_ANDROGEN_RESPONSE**	92	-0.5	**-2.0**	0	**6.90E-04**	0.002	2438
**HALLMARK_PROTEIN_SECRETION**	93	-0.5	**-1.9**	0	**0.002**	0.005	2470
**HALLMARK_FATTY_ACID_METABOLISM**	129	-0.5	**-1.8**	0.002	**0.001**	0.006	1911
**HALLMARK_GLYCOLYSIS**	178	-0.4	**-1.8**	0	**0.001**	0.006	1728
**HALLMARK_XENOBIOTIC_METABOLISM**	133	-0.4	**-1.8**	0	**0.002**	0.011	1308
**HALLMARK_OXIDATIVE_PHOSPHORYLATION**	197	-0.4	**-1.7**	0	**0.006**	0.035	3571
**HALLMARK_TNFA_SIGNALING_VIA_NFKB**	156	-0.4	**-1.7**	0	**0.006**	0.038	1332
**HALLMARK_EPITHELIAL_MESENCHYMAL_TRANSITION**	119	-0.4	**-1.6**	0.002	**0.018**	0.126	1194
**HALLMARK_MTORC1_SIGNALING**	192	-0.4	**-1.6**	0.003	**0.018**	0.14	2235
**HALLMARK_ESTROGEN_RESPONSE_LATE**	193	-0.4	**-1.5**	0.002	**0.024**	0.198	844
**HALLMARK_COAGULATION**	64	-0.4	**-1.5**	0.021	**0.030**	0.259	1078
**HALLMARK_ANGIOGENESIS**	23	-0.5	**-1.5**	0.053	**0.037**	0.319	2003
**HALLMARK_MYOGENESIS**	113	-0.4	**-1.4**	0.023	**0.064**	0.523	1016
**HALLMARK_ADIPOGENESIS**	172	-0.3	**-1.4**	0.016	**0.065**	0.548	1733
**HALLMARK_COMPLEMENT**	119	-0.3	**-1.4**	0.022	**0.067**	0.583	1682
**HALLMARK_IL2_STAT5_SIGNALING**	133	-0.3	**-1.4**	0.038	**0.080**	0.679	1728
**HALLMARK_KRAS_SIGNALING_DN**	76	-0.4	**-1.3**	0.065	**0.108**	0.781	893
**HALLMARK_INFLAMMATORY_RESPONSE**	96	-0.3	**-1.3**	0.062	**0.114**	0.809	1871
**HALLMARK_BILE_ACID_METABOLISM**	67	-0.4	**-1.3**	0.118	**0.140**	0.883	2232
**HALLMARK_PEROXISOME**	86	-0.3	**-1.3**	0.100	**0.136**	0.884	2371
**HALLMARK_UV_RESPONSE_DN**	124	-0.3	**-1.3**	0.075	**0.142**	0.906	1705
**HALLMARK_PI3K_AKT_MTOR_SIGNALING**	88	-0.3	**-1.2**	0.103	**0.158**	0.929	3475
**HALLMARK_P53_PATHWAY**	184	-0.3	**-1.2**	0.064	**0.152**	0.929	1396
**HALLMARK_TGF_BETA_SIGNALING**	51	-0.4	**-1.2**	0.164	**0.183**	0.963	1114
**HALLMARK_HEME_METABOLISM**	153	-0.3	**-1.2**	0.108	**0.192**	0.971	2244
HALLMARK_UNFOLDED_PROTEIN_RESPONSE	110	-0.3	-1.2	0.174	0.251	0.996	2916
HALLMARK_IL6_JAK_STAT3_SIGNALING	46	-0.3	-1.1	0.262	0.263	0.998	2243
HALLMARK_UV_RESPONSE_UP	136	-0.3	-1.1	0.247	0.309	0.998	2275
HALLMARK_APICAL_JUNCTION	133	-0.3	-1.0	0.401	0.476	1	1090
HALLMARK_NOTCH_SIGNALING	27	-0.3	-1.0	0.440	0.510	1	1479
HALLMARK_APICAL_SURFACE	26	-0.3	-1.0	0.426	0.505	1	1803
HALLMARK_SPERMATOGENESIS	62	-0.3	-0.9	0.606	0.709	1	2110
HALLMARK_HEDGEHOG_SIGNALING	26	-0.3	-0.9	0.598	0.700	1	1549
HALLMARK_MYC_TARGETS_V1	200	-0.2	-0.8	0.876	0.858	1	3764
HALLMARK_ALLOGRAFT_REJECTION	85	-0.2	-0.8	0.840	0.865	1	2156
HALLMARK_G2M_CHECKPOINT	198	-0.1	-0.5	1.000	0.998	1	2862

Supplementary Table S3 Gene Set Enrichment Analysis (GSEA)
Y537C Clones 1, 2 v MCF7-WT
Pathways UP

NAME	SIZE	ES	**NES**	NOM p-val	**FDR q-val**	FWER p-val	RANK AT MAX
**HALLMARK_EPITHELIAL_MESENCHYMAL_TRANSITION**	119	0.4	**1.6**	0.002	**0.049**	0.07	1685
**HALLMARK_APICAL_JUNCTION**	133	0.4	**1.5**	0.005	**0.068**	0.179	1736
**HALLMARK_HEDGEHOG_SIGNALING**	26	0.5	**1.4**	0.057	**0.108**	0.378	1684
**HALLMARK_ANGIOGENESIS**	23	0.5	**1.4**	0.089	**0.144**	0.568	1465
**HALLMARK_INTERFERON_GAMMA_RESPONSE**	141	0.3	**1.3**	0.044	**0.194**	0.761	1847
**HALLMARK_UV_RESPONSE_DN**	124	0.3	**1.3**	0.059	**0.183**	0.803	1161
**HALLMARK_ALLOGRAFT_REJECTION**	85	0.3	**1.2**	0.109	**0.225**	0.897	1991
**HALLMARK_TGF_BETA_SIGNALING**	51	0.3	**1.2**	0.189	**0.227**	0.931	1875
HALLMARK_INTERFERON_ALPHA_RESPONSE	82	0.3	1.2	0.197	0.264	0.966	1908
HALLMARK_WNT_BETA_CATENIN_SIGNALING	33	0.3	1.1	0.291	0.318	0.996	1440
HALLMARK_MITOTIC_SPINDLE	195	0.2	1.1	0.303	0.431	1	3015
HALLMARK_APICAL_SURFACE	26	0.3	1.1	0.373	0.398	1	1259
HALLMARK_SPERMATOGENESIS	62	0.3	0.9	0.559	0.619	1	1289
HALLMARK_G2M_CHECKPOINT	198	0.1	0.7	1.000	0.988	1	3231

Pathways DOWN

NAME	SIZE	ES	**NES**	NOM p-val	**FDR q-val**	FWER p-val	RANK AT MAX
**HALLMARK_OXIDATIVE_PHOSPHORYLATION**	197	-0.5	**-2.0**	0	**0.002**	0.002	3268
**HALLMARK_XENOBIOTIC_METABOLISM**	133	-0.5	**-1.9**	0	**0.001**	0.002	1241
**HALLMARK_ANDROGEN_RESPONSE**	92	-0.5	**-1.9**	0	**0.001**	0.003	2336
**HALLMARK_PROTEIN_SECRETION**	93	-0.5	**-1.8**	0	**0.001**	0.005	3236
**HALLMARK_FATTY_ACID_METABOLISM**	129	-0.4	**-1.8**	0	**0.002**	0.008	2160
**HALLMARK_HYPOXIA**	163	-0.4	**-1.7**	0	**0.009**	0.042	1441
**HALLMARK_ESTROGEN_RESPONSE_LATE**	193	-0.4	**-1.6**	0	**0.015**	0.079	983
**HALLMARK_GLYCOLYSIS**	178	-0.4	**-1.6**	0.002	**0.014**	0.085	1961
**HALLMARK_IL2_STAT5_SIGNALING**	133	-0.4	**-1.6**	0	**0.028**	0.183	1603
**HALLMARK_ADIPOGENESIS**	172	-0.4	**-1.5**	0.003	**0.030**	0.215	2182
**HALLMARK_KRAS_SIGNALING_UP**	101	-0.4	**-1.5**	0.011	**0.029**	0.228	1229
**HALLMARK_HEME_METABOLISM**	153	-0.4	**-1.5**	0	**0.029**	0.25	1617
**HALLMARK_MYC_TARGETS_V1**	200	-0.4	**-1.5**	0.003	**0.030**	0.273	4520
**HALLMARK_PEROXISOME**	86	-0.4	**-1.5**	0.018	**0.036**	0.341	1186
**HALLMARK_MTORC1_SIGNALING**	192	-0.4	**-1.5**	0.002	**0.039**	0.377	2813
**HALLMARK_ESTROGEN_RESPONSE_EARLY**	198	-0.3	**-1.4**	0.005	**0.069**	0.598	1013
**HALLMARK_BILE_ACID_METABOLISM**	67	-0.4	**-1.3**	0.065	**0.101**	0.76	1231
**HALLMARK_CHOLESTEROL_HOMEOSTASIS**	65	-0.4	**-1.3**	0.075	**0.101**	0.781	2420
**HALLMARK_KRAS_SIGNALING_DN**	76	-0.4	**-1.3**	0.062	**0.096**	0.783	834
**HALLMARK_MYC_TARGETS_V2**	58	-0.4	**-1.3**	0.085	**0.106**	0.829	3171
**HALLMARK_TNFA_SIGNALING_VIA_NFKB**	156	-0.3	**-1.3**	0.038	**0.103**	0.834	1560
**HALLMARK_UV_RESPONSE_UP**	136	-0.3	**-1.3**	0.064	**0.137**	0.921	1681
**HALLMARK_COAGULATION**	64	-0.3	**-1.2**	0.171	**0.217**	0.987	1359
HALLMARK_COMPLEMENT	119	-0.3	-1.2	0.155	0.255	0.993	1116
HALLMARK_MYOGENESIS	113	-0.3	-1.2	0.180	0.246	0.994	781
HALLMARK_P53_PATHWAY	184	-0.3	-1.1	0.220	0.322	1	1622
HALLMARK_PI3K_AKT_MTOR_SIGNALING	88	-0.3	-1.1	0.334	0.423	1	2888
HALLMARK_UNFOLDED_PROTEIN_RESPONSE	110	-0.3	-1.0	0.377	0.459	1	3554
HALLMARK_REACTIVE_OXYGEN_SPECIES_PATHWAY	45	-0.3	-1.0	0.469	0.566	1	2350
HALLMARK_INFLAMMATORY_RESPONSE	96	-0.3	-1.0	0.481	0.573	1	1439
HALLMARK_NOTCH_SIGNALING	27	-0.3	-1.0	0.468	0.555	1	1563
HALLMARK_IL6_JAK_STAT3_SIGNALING	46	-0.3	-1.0	0.502	0.543	1	2014
HALLMARK_APOPTOSIS	123	-0.2	-0.9	0.598	0.626	1	1449
HALLMARK_DNA_REPAIR	144	-0.2	-0.8	0.939	0.927	1	2362
HALLMARK_E2F_TARGETS	199	-0.1	-0.3	1.000	1.000	1	4035

Supplementary Table S4 Gene Set Enrichment Analysis (GSEA)
Y537N Clones 1-3 v MCF7-WT
Pathways UP

NAME	SIZE	ES	NES	NOM p-val	FDR q-val	FWER p-val	RANK AT MAX
**HALLMARK_INTERFERON_ALPHA_RESPONSE**	82	0.6	**2.3**	0	**0**	0	3027
**HALLMARK_INTERFERON_GAMMA_RESPONSE**	141	0.5	**2.1**	0	**0**	0	3071
**HALLMARK_ESTROGEN_RESPONSE_EARLY**	198	0.5	**2.0**	0	**0**	0	1805
**HALLMARK_APOPTOSIS**	123	0.5	**2.0**	0	**2.80E-04**	0.001	1870
**HALLMARK_EPITHELIAL_MESENCHYMAL_TRANSITION**	119	0.5	**1.9**	0	**4.19E-04**	0.002	1921
**HALLMARK_HYPOXIA**	163	0.4	**1.7**	0	**0.004**	0.024	1685
**HALLMARK_ESTROGEN_RESPONSE_LATE**	193	0.4	**1.7**	0	**0.004**	0.025	1898
**HALLMARK_TNFA_SIGNALING_VIA_NFKB**	156	0.4	**1.7**	0	**0.004**	0.033	2238
**HALLMARK_COAGULATION**	64	0.5	**1.7**	0.003	**0.007**	0.061	817
**HALLMARK_IL6_JAK_STAT3_SIGNALING**	46	0.5	**1.7**	0.006	**0.007**	0.065	2203
**HALLMARK_IL2_STAT5_SIGNALING**	133	0.4	**1.6**	0.001	**0.009**	0.088	1906
**HALLMARK_GLYCOLYSIS**	178	0.4	**1.6**	0	**0.008**	0.092	2928
**HALLMARK_REACTIVE_OXYGEN_SPECIES_PATHWAY**	45	0.5	**1.5**	0.017	**0.021**	0.234	2872
**HALLMARK_P53_PATHWAY**	184	0.4	**1.5**	0.001	**0.031**	0.334	1835
**HALLMARK_INFLAMMATORY_RESPONSE**	96	0.4	**1.5**	0.012	**0.035**	0.388	1399
**HALLMARK_COMPLEMENT**	119	0.4	**1.4**	0.024	**0.056**	0.57	2800
**HALLMARK_UV_RESPONSE_DN**	124	0.4	**1.4**	0.022	**0.057**	0.601	1771
**HALLMARK_NOTCH_SIGNALING**	27	0.5	**1.4**	0.068	**0.058**	0.623	2820
**HALLMARK_ALLOGRAFT_REJECTION**	85	0.3	**1.3**	0.082	**0.148**	0.937	2472
**HALLMARK_UV_RESPONSE_UP**	136	0.3	**1.3**	0.070	**0.161**	0.958	2624
HALLMARK_TGF_BETA_SIGNALING	51	0.3	1.2	0.236	0.317	0.999	2084
HALLMARK_XENOBIOTIC_METABOLISM	133	0.3	1.2	0.173	0.307	0.999	2299
HALLMARK_APICAL_SURFACE	26	0.4	1.1	0.265	0.353	1	601
HALLMARK_KRAS_SIGNALING_UP	101	0.3	1.1	0.221	0.348	1	1865
HALLMARK_HEME_METABOLISM	153	0.3	1.1	0.279	0.423	1	2605
HALLMARK_PEROXISOME	86	0.3	1.1	0.326	0.434	1	2417
HALLMARK_APICAL_JUNCTION	133	0.3	1.1	0.311	0.442	1	2100
HALLMARK_BILE_ACID_METABOLISM	67	0.3	1.1	0.344	0.452	1	2276
HALLMARK_ADIPOGENESIS	172	0.2	1.0	0.444	0.567	1	1978
HALLMARK_PI3K_AKT_MTOR_SIGNALING	88	0.3	1.0	0.460	0.573	1	3347
HALLMARK_ANDROGEN_RESPONSE	92	0.3	1.0	0.506	0.595	1	2362
HALLMARK_KRAS_SIGNALING_DN	76	0.2	0.9	0.713	0.846	1	2083
HALLMARK_ANGIOGENESIS	23	0.3	0.9	0.686	0.857	1	2158
HALLMARK_PROTEIN_SECRETION	93	0.2	0.9	0.738	0.833	1	2313
HALLMARK_MTORC1_SIGNALING	192	0.2	0.8	0.938	0.946	1	2577
HALLMARK_OXIDATIVE_PHOSPHORYLATION	197	0.2	0.7	0.996	1.000	1	2910
HALLMARK_UNFOLDED_PROTEIN_RESPONSE	110	0.2	0.6	0.999	0.994	1	2696

Pathways DOWN

NAME	SIZE	ES	NES	NOM p-val	FDR q-val	FWER p-val	RANK AT MAX
**HALLMARK_E2F_TARGETS**	199	-0.5	**-2.3**	0	**0**	0	3731
**HALLMARK_G2M_CHECKPOINT**	198	-0.4	**-2.2**	0	**0**	0	2706
**HALLMARK_CHOLESTEROL_HOMEOSTASIS**	65	-0.4	**-1.5**	0.006	**0.033**	0.117	2294
**HALLMARK_MYC_TARGETS_V1**	200	-0.3	**-1.4**	0	**0.063**	0.268	4032
**HALLMARK_MYC_TARGETS_V2**	58	-0.3	**-1.3**	0.103	**0.188**	0.67	3745
**HALLMARK_MYOGENESIS**	113	-0.3	**-1.2**	0.075	**0.197**	0.762	644
HALLMARK_MITOTIC_SPINDLE	195	-0.2	-1.2	0.079	0.269	0.9	2359
HALLMARK_HEDGEHOG_SIGNALING	26	-0.3	-1.0	0.363	0.530	0.998	34
HALLMARK_FATTY_ACID_METABOLISM	129	-0.2	-1.0	0.374	0.505	0.998	625
HALLMARK_WNT_BETA_CATENIN_SIGNALING	33	-0.3	-1.0	0.420	0.494	0.999	1793
HALLMARK_SPERMATOGENESIS	62	-0.2	-0.9	0.715	0.828	1	2196
HALLMARK_DNA_REPAIR	144	-0.2	-0.7	0.988	0.956	1	2556

Supplementary Table S5 Gene Set Enrichment Analysis (GSEA)
Y537S Clones 1-3 v MCF7-WT
Pathways UP

NAME	SIZE	ES	**NES**	NOM p-val	**FDR q-val**	FWER p-val	RANK AT MAX
**HALLMARK_INTERFERON_ALPHA_RESPONSE**	82	0.6	**2.4**	0	**0**	0	1720
**HALLMARK_INTERFERON_GAMMA_RESPONSE**	141	0.5	**2.2**	0	**0**	0	1720
**HALLMARK_ESTROGEN_RESPONSE_EARLY**	198	0.5	**2.1**	0	**0**	0	1863
**HALLMARK_ESTROGEN_RESPONSE_LATE**	193	0.4	**1.8**	0	**0.003**	0.014	1849
**HALLMARK_EPITHELIAL_MESENCHYMAL_TRANSITION**	119	0.4	**1.8**	0	**0.002**	0.014	2026
**HALLMARK_TNFA_SIGNALING_VIA_NFKB**	156	0.4	**1.6**	0.003	**0.011**	0.082	2199
**HALLMARK_ALLOGRAFT_REJECTION**	85	0.4	**1.6**	0.002	**0.015**	0.123	1973
**HALLMARK_P53_PATHWAY**	184	0.3	**1.5**	0.003	**0.044**	0.359	1325
**HALLMARK_WNT_BETA_CATENIN_SIGNALING**	33	0.4	**1.4**	0.055	**0.050**	0.434	2313
**HALLMARK_APICAL_JUNCTION**	133	0.3	**1.4**	0.020	**0.054**	0.494	1283
**HALLMARK_TGF_BETA_SIGNALING**	51	0.4	**1.4**	0.036	**0.057**	0.543	663
**HALLMARK_ANGIOGENESIS**	23	0.5	**1.4**	0.077	**0.054**	0.553	2522
**HALLMARK_COAGULATION**	64	0.3	**1.3**	0.064	**0.092**	0.789	1969
**HALLMARK_INFLAMMATORY_RESPONSE**	96	0.3	**1.3**	0.074	**0.131**	0.907	1973
**HALLMARK_IL6_JAK_STAT3_SIGNALING**	46	0.4	**1.3**	0.140	**0.136**	0.933	2348
**HALLMARK_APOPTOSIS**	123	0.3	**1.2**	0.122	**0.201**	0.99	2308
**HALLMARK_APICAL_SURFACE**	26	0.4	**1.2**	0.210	**0.192**	0.991	2749
HALLMARK_KRAS_SIGNALING_DN	76	0.3	**1.1**	0.237	0.293	0.998	1912
HALLMARK_UV_RESPONSE_DN	124	0.3	**1.1**	0.271	0.325	1	2209
HALLMARK_HEDGEHOG_SIGNALING	26	0.4	**1.1**	0.372	0.328	1	2601
HALLMARK_MITOTIC_SPINDLE	195	0.2	**1.0**	0.388	0.445	1	2920
HALLMARK_NOTCH_SIGNALING	27	0.3	**0.9**	0.620	0.718	1	2027
HALLMARK_REACTIVE_OXYGEN_SPECIES_PATHWAY	45	0.2	**0.8**	0.840	0.893	1	1075

Pathways DOWN

NAME	SIZE	ES	**NES**	NOM p-val	**FDR q-val**	FWER p-val	RANK AT MAX
**HALLMARK_PROTEIN_SECRETION**	93	-0.4	**-1.9**	0	**0.005**	0.004	3674
**HALLMARK_ANDROGEN_RESPONSE**	92	-0.4	**-1.9**	0	**0.003**	0.005	1274
**HALLMARK_FATTY_ACID_METABOLISM**	129	-0.4	**-1.9**	0	**0.002**	0.005	2466
**HALLMARK_OXIDATIVE_PHOSPHORYLATION**	197	-0.4	**-1.8**	0	**0.004**	0.011	2741
**HALLMARK_CHOLESTEROL_HOMEOSTASIS**	65	-0.4	**-1.7**	0.002	**0.006**	0.024	1553
**HALLMARK_XENOBIOTIC_METABOLISM**	133	-0.4	**-1.7**	0	**0.008**	0.034	905
**HALLMARK_PEROXISOME**	86	-0.4	**-1.6**	0	**0.023**	0.116	1288
**HALLMARK_MTORC1_SIGNALING**	192	-0.3	**-1.5**	0	**0.026**	0.15	2405
**HALLMARK_KRAS_SIGNALING_UP**	101	-0.3	**-1.5**	0.024	**0.030**	0.189	967
**HALLMARK_GLYCOLYSIS**	178	-0.3	**-1.5**	0.005	**0.029**	0.204	2114
**HALLMARK_HYPOXIA**	163	-0.3	**-1.4**	0.008	**0.049**	0.348	1327
**HALLMARK_HEME_METABOLISM**	153	-0.3	**-1.4**	0.012	**0.049**	0.371	1696
**HALLMARK_ADIPOGENESIS**	172	-0.3	**-1.4**	0.002	**0.047**	0.379	2710
**HALLMARK_MYOGENESIS**	113	-0.3	**-1.4**	0.031	**0.077**	0.569	1158
**HALLMARK_BILE_ACID_METABOLISM**	67	-0.3	**-1.4**	0.046	**0.073**	0.573	1274
**HALLMARK_IL2_STAT5_SIGNALING**	133	-0.3	**-1.2**	0.103	**0.192**	0.93	1195
**HALLMARK_MYC_TARGETS_V1**	200	-0.2	**-1.2**	0.134	**0.248**	0.969	3281
HALLMARK_PI3K_AKT_MTOR_SIGNALING	88	-0.3	**-1.2**	0.196	0.255	0.977	2114
HALLMARK_UNFOLDED_PROTEIN_RESPONSE	110	-0.3	**-1.1**	0.205	0.293	0.99	3447
HALLMARK_COMPLEMENT	119	-0.2	**-1.0**	0.326	0.445	0.999	1132
HALLMARK_UV_RESPONSE_UP	136	-0.2	**-1.0**	0.462	0.554	1	2257
HALLMARK_E2F_TARGETS	199	-0.2	**-1.0**	0.542	0.608	1	2861
HALLMARK_SPERMATOGENESIS	62	-0.2	**-1.0**	0.518	0.605	1	2723
HALLMARK_G2M_CHECKPOINT	198	-0.2	**-0.9**	0.885	0.853	1	2943
HALLMARK_DNA_REPAIR	144	-0.2	**-0.7**	0.982	0.987	1	2676
HALLMARK_MYC_TARGETS_V2	58	-0.2	**-0.7**	0.977	0.987	1	3256

Supplementary Table S6 Gene Set Enrichment Analysis (GSEA)
D538G Clones 1-4 v MCF7-WT
Pathways UP

NAME	SIZE	ES	**NES**	NOM p-val	**FDR q-val**	FWER p-val	RANK AT MAX
**HALLMARK_INTERFERON_ALPHA_RESPONSE**	82	0.7	**2.8**	0	**0**	0	1755
**HALLMARK_INTERFERON_GAMMA_RESPONSE**	141	0.6	**2.4**	0	**0**	0	1670
**HALLMARK_ALLOGRAFT_REJECTION**	85	0.4	**1.5**	0.013	**0.044**	0.154	2358
**HALLMARK_ESTROGEN_RESPONSE_EARLY**	198	0.3	**1.4**	0.018	**0.114**	0.43	1282
**HALLMARK_HEDGEHOG_SIGNALING**	26	0.4	**1.4**	0.071	**0.119**	0.527	1791
**HALLMARK_EPITHELIAL_MESENCHYMAL_TRANSITION**	119	0.3	**1.3**	0.022	**0.103**	0.543	580
**HALLMARK_APICAL_JUNCTION**	133	0.3	**1.3**	0.056	**0.126**	0.68	2105
**HALLMARK_P53_PATHWAY**	184	0.3	**1.2**	0.047	**0.187**	0.848	1547
HALLMARK_APOPTOSIS	123	0.3	**1.2**	0.146	0.288	0.961	2292
HALLMARK_TGF_BETA_SIGNALING	51	0.3	**1.1**	0.238	0.273	0.966	770
HALLMARK_INFLAMMATORY_RESPONSE	96	0.3	**1.1**	0.199	0.266	0.972	2175
HALLMARK_IL6_JAK_STAT3_SIGNALING	46	0.3	**1.1**	0.311	0.307	0.987	1491
HALLMARK_ANGIOGENESIS	23	0.3	**0.9**	0.526	0.675	1	1373
HALLMARK_UNFOLDED_PROTEIN_RESPONSE	110	0.2	**0.9**	0.739	0.797	1	566
HALLMARK_MYC_TARGETS_V2	58	0.2	**0.7**	0.969	0.979	1	3388

Pathways DOWN

NAME	SIZE	ES	**NES**	NOM p-val	**FDR q-val**	FWER p-val	RANK AT MAX
**HALLMARK_PROTEIN_SECRETION**	93	-0.5	**-1.9**	0	**0.001**	0.001	2063
**HALLMARK_FATTY_ACID_METABOLISM**	129	-0.5	**-1.9**	0	**0.003**	0.005	2688
**HALLMARK_HYPOXIA**	163	-0.5	**-1.8**	0	**0.002**	0.007	1658
**HALLMARK_XENOBIOTIC_METABOLISM**	133	-0.5	**-1.8**	0	**0.002**	0.007	1231
**HALLMARK_GLYCOLYSIS**	178	-0.4	**-1.8**	0	**0.002**	0.01	1960
**HALLMARK_OXIDATIVE_PHOSPHORYLATION**	197	-0.4	**-1.8**	0	**0.003**	0.014	3199
**HALLMARK_ANDROGEN_RESPONSE**	92	-0.5	**-1.7**	0.002	**0.004**	0.024	1642
**HALLMARK_ADIPOGENESIS**	172	-0.4	**-1.6**	0.003	**0.018**	0.114	2853
**HALLMARK_KRAS_SIGNALING_UP**	101	-0.4	**-1.6**	0.003	**0.023**	0.162	1255
**HALLMARK_HEME_METABOLISM**	153	-0.4	**-1.6**	0.002	**0.021**	0.165	1705
**HALLMARK_IL2_STAT5_SIGNALING**	133	-0.4	**-1.5**	0.005	**0.029**	0.242	1354
**HALLMARK_KRAS_SIGNALING_DN**	76	-0.4	**-1.5**	0.020	**0.035**	0.308	1291
**HALLMARK_CHOLESTEROL_HOMEOSTASIS**	65	-0.4	**-1.5**	0.024	**0.041**	0.383	2707
**HALLMARK_NOTCH_SIGNALING**	27	-0.5	**-1.4**	0.084	**0.083**	0.648	1117
**HALLMARK_PEROXISOME**	86	-0.4	**-1.4**	0.049	**0.088**	0.692	1594
**HALLMARK_MTORC1_SIGNALING**	192	-0.3	**-1.3**	0.023	**0.108**	0.786	2514
**HALLMARK_BILE_ACID_METABOLISM**	67	-0.4	**-1.3**	0.069	**0.104**	0.794	2699
**HALLMARK_UV_RESPONSE_DN**	124	-0.3	**-1.3**	0.033	**0.105**	0.819	1977
**HALLMARK_ESTROGEN_RESPONSE_LATE**	193	-0.3	**-1.2**	0.096	**0.206**	0.972	1194
HALLMARK_COAGULATION	64	-0.3	**-1.2**	0.183	0.277	0.999	1080
HALLMARK_COMPLEMENT	119	-0.3	**-1.2**	0.175	0.271	0.999	1772
HALLMARK_MYOGENESIS	113	-0.3	**-1.1**	0.215	0.373	1	2170
HALLMARK_TNFA_SIGNALING_VIA_NFKB	156	-0.3	**-1.1**	0.247	0.404	1	1426
HALLMARK_UV_RESPONSE_UP	136	-0.3	**-1.1**	0.292	0.399	1	1890
HALLMARK_PI3K_AKT_MTOR_SIGNALING	88	-0.3	**-1.0**	0.362	0.488	1	2037
HALLMARK_SPERMATOGENESIS	62	-0.3	**-1.0**	0.490	0.639	1	2539
HALLMARK_WNT_BETA_CATENIN_SIGNALING	33	-0.3	**-0.9**	0.606	0.854	1	246
HALLMARK_MYC_TARGETS_V1	200	-0.2	**-0.8**	0.915	1.000	1	2772
HALLMARK_MITOTIC_SPINDLE	195	-0.2	**-0.8**	0.943	1.000	1	2105
HALLMARK_DNA_REPAIR	144	-0.2	**-0.8**	0.951	1.000	1	2800
HALLMARK_APICAL_SURFACE	26	-0.3	**-0.7**	0.845	1.000	1	1297
HALLMARK_REACTIVE_OXYGEN_SPECIES_PATHWAY	45	-0.2	**-0.7**	0.943	1.000	1	2778
HALLMARK_G2M_CHECKPOINT	198	-0.1	**-0.5**	1.000	1.000	1	2883
HALLMARK_E2F_TARGETS	199	-0.1	**-0.4**	1.000	1.000	1	2596

Supplementary Table S7
Differential genes common to all ESR1 mutations

Genes	Cluster
RGAG4	UP
PRKAR2B	UP
RAB3IL1	UP
DLC1	UP
TOX2	UP
B3GNT4	UP
ZNF385D	UP
ZNF417	UP
MUC3A	UP
PALM3	UP
RP11-321G12.1	UP
CLIP4	UP
FOSL1	UP
MAG	UP
CD22	UP
OLFML3	UP
PROCR	UP
SMARCD3	UP
KNDC1	UP
ZNF416	UP
SLC4A3	UP
FAM19A5	UP
COL5A1	UP
C1orf95	UP
KCNMA1	UP
PTGER3	UP
CDH2	UP
PTGER2	UP
CRABP1	UP
RND2	UP
GULP1	UP
PDZK1	UP
RERG	UP
SULT1A1	UP
NUAK2	UP
C5AR2	UP
APOL3	UP
IFI35	UP
UBE2L6	UP
OAS2	UP
PARP10	UP
PSMB8	UP
ACTG2	UP
PSMB9	UP
TAP2	UP
KCNQ2	UP
MAP1B	UP
RP11-556I14.1	UP
MGP	UP
AL356737.1	UP
LANCL3	UP
FRAS1	UP
PMP22	UP
RP11-662G23.1	UP
SOCS1	UP
ANXA3	UP
OSGIN1	UP
CCDC85A	UP
AC007743.1	UP
CRABP2	UP
GPRC5C	UP
CA12	UP
NPY1R	UP
MAGEE1	UP
HCN4	UP
DLGAP3	UP
SEMA3D	UP
HMCN1	UP
AC005256.1	UP
PYCARD	UP
MTMR7	UP
GPC6	UP
COL3A1	UP
MTCL1	UP
FHL1	UP
GALNTL6	UP
NRG3	UP
PPP1R1C	UP
EPHA6	UP
NTSR1	UP
AC011747.4	UP
PSTPIP2	UP
NPR3	UP
LINC00052	UP

Genes	Cluster
AP000439.3	DOWN
MYZAP	DOWN
FSTL4	DOWN
MT1X	DOWN
RASD1	DOWN
KRT86	DOWN
CA2	DOWN
ACSL4	DOWN
NRP2	DOWN
RP11-466H18.1	DOWN
ITPRIPL2	DOWN
MUC1	DOWN
ATP8A1	DOWN
KRT81	DOWN
SSPN	DOWN
PLLP	DOWN
SEMA5A	DOWN
SCARA3	DOWN
C9orf152	DOWN
CEMIP	DOWN
PTPRN2	DOWN
VAT1L	DOWN
CRAT	DOWN
CEACAM6	DOWN
MDFIC	DOWN
ERGIC1	DOWN
PTPRU	DOWN
PP14571	DOWN
PLXNA2	DOWN
CYP2T2P	DOWN
MUC5AC	DOWN
SLPI	DOWN
CYP2S1	DOWN
PTGES	DOWN
GDPD3	DOWN
APCDD1	DOWN
C2orf54	DOWN
GALNT12	DOWN
FOXD2	DOWN
HSD17B11	DOWN
RAPGEF3	DOWN
SLC9A2	DOWN
WNT11	DOWN
ABCG2	DOWN
EHD2	DOWN

GATA4	UP
PCDH17	UP
FBN2	UP
SLC30A3	UP
FAM131C	UP
UBASH3B	UP
PLXNA4	UP
SGPP2	UP
NFIA	UP
MYO10	UP
LIMK1	UP
ADA	UP
DMTN	UP
CLIP2	UP
RAB31	UP
NDRG4	UP
NXN	UP
FAM171A1	UP
SYT8	UP
THEMIS2	UP
FBXO6	UP
CDSN	UP
LRRC61	UP
ZBED6CL	UP
PGBD5	UP
NUPR1	UP
GABBR1	UP
CYP1A1	UP
ZNF268	UP
COL6A1	UP
DIRAS1	UP
COL6A2	UP
PIK3CD	UP
GPNMB	UP
S100A7	UP
ARMCX2	UP
LPAR1	UP
SLC22A31	UP
RIN3	UP
ATP8A2	UP
SMOC2	UP
SDK2	UP
SMTNL2	UP
ITGA2B	UP
FBXO2	UP
C15orf59	UP
TRPV3	UP
SLC47A1	UP
GRIK3	UP
PDLIM3	UP
LRG1	UP
IL24	UP
FAIM3	UP
RP11-564A8.4	UP
IL20	UP
C4A	UP
CFB	UP
SLC25A18	UP
GPR68	UP
ARTN	UP
CPPED1	UP
PREX1	UP
B4GALNT1	UP
ADORA1	UP
HSPB8	UP
RTN4RL1	UP
CISH	UP
APLP1	UP
CHST8	UP
FHL2	UP
SDC2	UP
RHOBTB1	UP
LCN2	UP
PXMP4	UP
RILP	UP
RP11-64B16.2	UP
BMP2	UP
ATP2B4	UP
SELM	UP
CPQ	UP
PTK6	UP
ARHGAP4	UP
UBE2QL1	UP
TCIRG1	UP
IFI30	UP
SLFN5	UP
CAMK1	UP
MESP2	UP

Supplementary Table S8 Gene Set Enrichment Analysis (GSEA)
Differential genes common to all ESR1 mutations
Pathways UP

NAME	# Genes in Gene Set (K)	# Genes in Overlap (k)	k/K	FDR q-val	Genes										
HALLMARK_UV_RESPONSE_DN	144	11	0.0764	1.43E-10	DLC1	FHL2	ATP2B4	PRKAR2B	COL3A1	PMP22	SDC2	LPAR1	KCNMA1	MAP1B	PIK3CD
HALLMARK_ESTROGEN_RESPONSE_EARLY	200	11	0.055	1.68E-09	DLC1	FHL2	CISH	PDLIM3	HSPB8	RAB31	CA12	NPY1R	PDZK1	OLFML3	FAIM3
HALLMARK_ESTROGEN_RESPONSE_LATE	200	11	0.055	1.68E-09	ATP2B4	PRKAR2B	CISH	PDLIM3	HSPB8	RAB31	CA12	NPY1R	PDZK1	PTGER3	CHST8
HALLMARK_EPITHELIAL_MESENCHYMAL_TRANSITION	200	9	0.045	3.80E-07	COL3A1	PMP22	APLP1	COL6A2	COL5A1	CDH2	FBN2	MGP	MAGEE1		
HALLMARK_INTERFERON_GAMMA_RESPONSE	200	8	0.04	4.45E-06	IFI30	PSMB8	UBE2L6	PSMB9	IFI35	SOCS1	CFB	OAS2			
HALLMARK_INTERFERON_ALPHA_RESPONSE	97	6	0.0619	8.54E-06	IFI30	PSMB8	UBE2L6	PSMB9	IFI35	PROCR					
HALLMARK_P53_PATHWAY	200	7	0.035	4.10E-05	IFI30	SOCS1	PROCR	BMP2	NUPR1	ADA	OSGIN1				
HALLMARK_IL2_STAT5_SIGNALING	200	6	0.03	4.02E-04	CISH	APLP1	SOCS1	BMP2	PTGER2	COL6A1					
HALLMARK_UV_RESPONSE_UP	158	4	0.0253	1.16E-02	PDLIM3	APLP1	SULT1A1	CYP1A1							
HALLMARK_KRAS_SIGNALLING_UP	200	4	0.02	2.20E-02	PSMB8	CFB	BMP2	GPNMB							

Pathways DOWN

NAME	# Genes in Gene Set (K)	# Genes in Overlap (k)	k/K	FDR q-val	Genes			
HALLMARK_FATTY_ACID_METABOLISM	158	4	0.0253	6.17E-04	CRAT	HSD17B11	ACSL4	CA2
HALLMARK_PEROXISOME	104	3	0.0288	2.82E-03	CRAT	HSD17B11	ACSL4	
HALLMARK_XENOBIOTIC_METABOLISM	200	3	0.015	1.28E-02	CA2	PTGES	CYP2S1	

Supplementary Table S9 Gene Signatures. 136 UP and 42 DOWN genes
(In bold are the genes also present in the smaller gene set used in Signature 3)

UP Genes

EnsembleID	Gene	baseMean	Log2 FC rel MCF7	padj
ENSG00000106683	LIMK1	2127.612527	1.278340889	7.0386E-112
**ENSG00000162599**	**NFIA**	**532.4713641**	**3.658160963**	**4.68938E-93**
ENSG00000168461	RAB31	2855.805016	1.56473372	4.37688E-43
ENSG00000103490	PYCARD	1522.982631	1.378965893	6.42362E-42
ENSG00000196839	ADA	389.3664252	1.858394217	1.53942E-41
**ENSG00000164128**	**NPY1R**	**1490.41942**	**3.898461046**	**4.22397E-41**
**ENSG00000162894**	**FAIM3**	**253.0833015**	**4.268865966**	**5.7856E-40**
**ENSG00000158856**	**DMTN**	**521.1526385**	**1.931654325**	**4.84692E-39**
**ENSG00000167994**	**RAB3IL1**	**330.4408154**	**2.15144047**	**2.03624E-37**
**ENSG00000074410**	**CA12**	**6398.835475**	**1.970130223**	**8.69857E-37**
ENSG00000140961	OSGIN1	1501.526598	1.582380057	1.95531E-34
ENSG00000152137	HSPB8	2929.518789	4.382370696	2.98887E-33
ENSG00000135454	B4GALNT1	692.1763225	1.856264242	7.9365E-33
ENSG00000198832	SELM	330.6520524	1.609825832	1.41596E-31
ENSG00000170412	GPRC5C	1155.720125	1.530014104	5.5236E-31
ENSG00000164741	DLC1	1066.688345	3.258643697	1.05856E-27
**ENSG00000003987**	**MTMR7**	**106.2107024**	**1.847210872**	**4.14666E-27**
ENSG00000142173	COL6A2	332.8011361	2.831375691	3.07402E-25
ENSG00000124126	PREX1	28229.23249	2.022133021	8.16861E-25
**ENSG00000162891**	**IL20**	**268.9226145**	**5.82590076**	**1.20244E-24**
ENSG00000138622	HCN4	90.918415	2.092038918	2.08178E-24
ENSG00000138772	ANXA3	1080.585241	1.767148766	4.09661E-24
ENSG00000185338	SOCS1	175.2183319	2.353513377	4.54724E-24
**ENSG00000136574**	**GATA4**	**89.24544754**	**6.003473563**	**1.41931E-23**
ENSG00000103381	CPPED1	401.9548423	1.297277143	2.18522E-23
ENSG00000196502	SULT1A1	337.2904752	2.732281982	1.28644E-22
ENSG00000162892	IL24	130.6555232	5.897365182	1.51481E-22
ENSG00000185924	RTN4RL1	1417.530617	1.287766197	2.29215E-22
ENSG00000083817	ZNF416	80.63248364	1.688059444	2.95122E-22
ENSG00000109099	PMP22	2050.349813	2.911951934	4.93698E-22
ENSG00000163545	NUAK2	181.8794595	2.6403374	4.99507E-22
ENSG00000198934	MAGEE1	111.4340339	1.707286537	6.02696E-22
ENSG00000058668	ATP2B4	575.4466682	1.66243861	6.25253E-22
ENSG00000216490	IFI30	1255.787544	2.014343109	9.24181E-22
ENSG00000134830	C5AR2	702.6625095	2.13013705	3.16534E-21
ENSG00000104324	CPQ	88.71818893	2.075577276	5.19603E-21
ENSG00000108830	RND2	111.0205214	2.602415963	8.45056E-21
ENSG00000187867	PALM3	286.9622974	1.634158849	1.32394E-20
ENSG00000116774	OLFML3	91.35211444	4.453810058	1.76925E-20
ENSG00000176046	NUPR1	560.1116359	5.079101048	2.20025E-20
ENSG00000183098	GPC6	974.0952546	1.587925807	2.27593E-20
ENSG00000082014	SMARCD3	321.1977448	1.595535793	9.54073E-20
ENSG00000130635	COL5A1	726.0561861	3.757664793	1.35954E-19
ENSG00000168502	MTCL1	668.8199598	1.769698819	1.40855E-19
ENSG00000168542	COL3A1	1779.243591	5.603891967	1.58289E-19
ENSG00000171236	LRG1	229.8298961	2.634756959	2.72086E-19
ENSG00000114923	SLC4A3	158.8096075	1.93164607	5.36758E-19
ENSG00000114737	CISH	568.1336586	1.437284441	1.05999E-18
ENSG00000105290	APLP1	425.1315479	1.237448742	1.16243E-18
ENSG00000145555	MYO10	1014.086034	2.113906792	1.61797E-18
ENSG00000105695	MAG	85.60297137	4.054950507	2.75924E-18
ENSG00000242732	RGAG4	77.23046491	1.743952363	5.27771E-18
ENSG00000176383	B3GNT4	142.2799003	1.411629827	6.9724E-18
ENSG00000005249	PRKAR2B	969.0399055	2.257031466	8.79545E-18
ENSG00000127399	LRRC61	308.3826763	2.196115977	1.17238E-17
ENSG00000176490	DIRAS1	168.0146684	2.604508851	1.29202E-17
ENSG00000134533	RERG	773.5594958	2.714208168	1.84794E-17
ENSG00000110719	TCIRG1	306.8046034	4.066503214	1.88648E-17
ENSG00000215218	UBE2QL1	171.4403778	3.537923422	3.39105E-17
ENSG00000188707	ZBED6CL	157.8029195	2.412826858	4.14866E-17
ENSG00000116544	DLGAP3	93.47815442	1.609862886	4.47317E-17
ENSG00000163082	SGPP2	72.88461661	1.972186389	5.23506E-17
ENSG00000124191	TOX2	259.9010283	1.619229529	6.6481E-17
ENSG00000103034	NDRG4	416.8783589	1.133800587	6.75731E-17
ENSG00000111341	MGP	3761.126017	3.642897078	6.88907E-17
ENSG00000143320	CRABP2	7466.121489	1.720599361	1.03187E-16
ENSG00000156587	UBE2L6	591.9008996	2.795879926	1.45422E-16
ENSG00000163485	ADORA1	653.980531	2.56347304	2.45284E-16
ENSG00000166426	CRABP1	789.2664028	2.108086533	7.43267E-16
ENSG00000101213	PTK6	205.8291632	2.703074023	1.70079E-15
ENSG00000148468	FAM171A1	144.219129	1.819957316	1.87388E-15
**ENSG00000188176**	**SMTNL2**	**96.65591498**	**3.258438859**	**2.11013E-15**
ENSG00000177614	PGBD5	184.0215077	2.580558807	2.60351E-15
ENSG00000101000	PROCR	79.01550583	1.848144105	5.23178E-15
ENSG00000134072	CAMK1	140.780296	1.751114949	6.2774E-15
ENSG00000125384	PTGER2	73.82703141	2.218319454	6.2774E-15
ENSG00000101417	PXMP4	139.7733747	1.247391588	6.82117E-15
ENSG00000022267	FHL1	543.3543977	1.55474262	1.02849E-14
ENSG00000144366	GULP1	510.0171758	2.436067722	1.30157E-14
ENSG00000169439	SDC2	1677.528906	2.344697157	1.36711E-14
ENSG00000163873	GRIK3	207.2086256	3.123154214	1.45957E-14
ENSG00000116661	FBXO2	134.3644036	3.645195213	2.07196E-14
ENSG00000167705	RILP	101.4715931	1.22280897	2.46073E-14
ENSG00000174827	PDZK1	1293.272299	2.548036462	4.74794E-14
ENSG00000069188	SDK2	146.2501159	2.714114379	5.74919E-14
ENSG00000090612	ZNF268	267.5240098	1.283738857	6.04489E-14
ENSG00000115641	FHL2	1463.655594	1.481971195	6.04489E-14
ENSG00000138829	FBN2	68.89859595	2.818892001	6.83622E-14
ENSG00000116663	FBXO6	125.3877431	2.999597636	7.96597E-14
ENSG00000124302	CHST8	181.2170044	1.816280265	8.08999E-14
ENSG00000153993	SEMA3D	129.4650703	3.820805821	1.50399E-13
ENSG00000055813	CCDC85A	147.2658625	3.208076064	4.28687E-13
ENSG00000152229	PSTPIP2	288.5702754	2.546407252	5.52994E-13
ENSG00000115194	SLC30A3	218.2079403	1.468297801	6.72839E-13
ENSG00000178685	PARP10	478.2799923	3.817222585	8.57239E-13
ENSG00000106665	CLIP2	277.7625572	1.281467167	9.90843E-13
ENSG00000171798	KNDC1	318.5109586	1.876953843	1.19262E-12
ENSG00000068079	IFI35	271.0133463	2.070528791	1.32033E-12
ENSG00000072422	RHOBTB1	261.5424315	1.667075958	1.61649E-12
ENSG00000171608	PIK3CD	75.34895838	2.52587589	2.01359E-12
ENSG00000185519	FAM131C	112.3420027	1.357906961	2.94511E-12
ENSG00000205363	C15orf59	153.1068128	4.355653503	3.31687E-12
ENSG00000101188	NTSR1	48.46696606	2.09214754	1.00318E-11
ENSG00000142494	SLC47A1	59.39261327	1.84522202	1.4176E-11
ENSG00000204681	GABBR1	60.20297937	1.793543812	1.61448E-11
ENSG00000154553	PDLIM3	89.10035413	2.846355798	1.70515E-11
ENSG00000167693	NXN	261.2182148	1.353450891	2.23556E-11
ENSG00000219438	FAM19A5	94.4973651	2.781628191	2.85808E-11
ENSG00000154127	UBASH3B	125.2288803	1.539939321	3.16078E-11
ENSG00000166750	SLFN5	231.5447269	2.261349734	4.37142E-11
ENSG00000113389	NPR3	272.162725	2.287148716	5.89564E-11
ENSG00000142156	COL6A1	247.3638466	1.854688347	1.36195E-10
ENSG00000143341	HMCN1	2268.205073	3.067057548	1.57326E-10
ENSG00000089820	ARHGAP4	150.6553979	1.682524551	2.46667E-10
ENSG00000156113	KCNMA1	67.60957989	1.80442893	5.07462E-10
ENSG00000115295	CLIP4	66.7188783	2.755791402	6.11631E-10
ENSG00000163017	ACTG2	168.2034337	3.077171887	6.6373E-10
ENSG00000131711	MAP1B	106.9580821	1.696856728	3.91465E-09
ENSG00000140465	CYP1A1	164.2657206	2.567604864	3.91465E-09
ENSG00000012124	CD22	83.84841342	2.282878998	4.51663E-09
ENSG00000117407	ARTN	84.97991786	2.102980288	4.89938E-09
ENSG00000240065	PSMB9	166.091829	2.208824405	1.04405E-08
ENSG00000100599	RIN3	160.6190064	1.883993245	1.21404E-08
ENSG00000169894	MUC3A	118.5985382	1.824113799	1.4169E-08
ENSG00000138759	FRAS1	492.623519	1.760587193	2.15431E-08
ENSG00000175592	FOSL1	155.6831647	1.427930143	3.74359E-08
ENSG00000149043	SYT8	60.79391952	1.741382027	3.78992E-08
ENSG00000119714	GPR68	476.8409916	1.358442955	7.30374E-08
ENSG00000173480	ZNF417	87.37136504	1.271537304	2.23793E-07
ENSG00000050628	PTGER3	148.1614257	2.103630641	8.76815E-07
ENSG00000243649	CFB	60.76965275	1.368039776	1.31112E-06
ENSG00000204267	TAP2	1208.304467	1.145960288	2.27721E-06
ENSG00000118946	PCDH17	329.7931541	2.78646295	3.35729E-06
ENSG00000080224	EPHA6	92.46287047	1.583919245	7.62135E-06
ENSG00000185737	NRG3	54.42340341	1.387323256	9.07331E-06
ENSG00000221866	PLXNA4	171.5474725	1.402242552	0.000128205

DOWN Genes

EnsembleID	Gene	baseMean	Log2 FC rel MCF7	padj
ENSG00000170442	KRT86	1685.903155	-3.860351308	2.3152E-86
ENSG00000113719	ERGIC1	8005.763442	-1.574623721	1.7433E-45
ENSG00000187193	MT1X	1071.208428	-2.002054989	7.6214E-36
ENSG00000119514	GALNT12	145.8337011	-2.145887408	1.6691E-30
ENSG00000060656	PTPRU	1682.318215	-1.364388829	1.5052E-29
ENSG00000218416	PP14571	165.6171265	-4.044577827	3.715E-25
ENSG00000186564	FOXD2	132.9391961	-1.221627532	6.3885E-25
ENSG00000108551	RASD1	236.5519776	-2.784130081	3.151E-24
ENSG00000115616	SLC9A2	391.4984557	-1.513619045	1.4684E-23
ENSG00000103888	CEMIP	653.5399667	-3.253501727	3.22E-20
ENSG00000148344	PTGES	646.6722936	-2.401177287	5.4482E-19
ENSG00000079337	RAPGEF3	79.41079191	-1.792884505	1.015E-18
ENSG00000167600	CYP2S1	112.4477159	-2.068139873	5.2351E-17
ENSG00000053108	FSTL4	87.85505209	-2.028932105	5.7084E-17
ENSG00000102934	PLLP	260.4319289	-2.296944539	1.8982E-16
ENSG00000168077	SCARA3	329.8696968	-2.07386431	5.2521E-16
ENSG00000076356	PLXNA2	175.1479982	-2.560026651	3.5354E-15
ENSG00000263155	MYZAP	89.19365433	-1.884420946	3.8732E-15
ENSG00000095321	CRAT	972.8517156	-3.599470171	4.7299E-15
ENSG00000118257	NRP2	58.16658071	-2.552448934	1.5171E-14
ENSG00000102886	GDPD3	280.1910424	-1.52230047	9.7582E-14
ENSG00000068366	ACSL4	89.16323194	-1.984106529	2.5345E-13
ENSG00000155093	PTPRN2	80.18122922	-2.782900141	4.5507E-13
ENSG00000024422	EHD2	633.2698534	-1.45596174	1.2445E-12
ENSG00000104267	CA2	1160.394663	-2.137360098	1.0712E-11
ENSG00000118777	ABCG2	1471.121623	-1.803114285	2.6305E-11
ENSG00000188959	C9orf152	131.3944869	-3.179049026	9.1694E-11
ENSG00000124406	ATP8A1	92.87546724	-2.083552827	1.5819E-10
ENSG00000205730	ITPRIPL2	709.0352109	-1.529274914	1.6064E-10
ENSG00000172478	C2orf54	121.6387876	-2.051652285	1.7447E-10
ENSG00000198189	HSD17B11	225.4450055	-1.876309666	4.7583E-09
ENSG00000124107	SLPI	46.28453874	-2.805459126	6.4816E-09
ENSG00000085741	WNT11	84.09665589	-2.632975005	7.2711E-09
ENSG00000135272	MDFIC	67.93107303	-3.343350023	2.5608E-08
ENSG00000123096	SSPN	113.5809034	-2.276205284	1.1193E-07
ENSG00000154856	APCDD1	157.7442664	-2.088158902	1.1931E-07
ENSG00000205426	KRT81	37957.63791	-2.359226179	4.2321E-07
ENSG00000112902	SEMA5A	194.1465779	-2.540367333	6.5665E-07
ENSG00000086548	CEACAM6	3477.603582	-3.719888077	7.2142E-07
ENSG00000171724	VAT1L	124.4816725	-2.639713686	7.7635E-07
ENSG00000215182	MUC5AC	801.6058046	-3.280056553	2.9603E-06
ENSG00000185499	MUC1	995.0771973	-2.037126196	0.00016265

Supplementary Table S10 Gene Signature 3

UP Genes

EnsembleID	Gene	baseMean	Log2 FC rel MCF7	padj
ENSG00000162599	NFIA	532.4713641	3.658160963	4.68938E-93
ENSG00000164128	NPY1R	1490.41942	3.898461046	4.22397E-41
ENSG00000162894	FAIM3	253.0833015	4.268865966	5.7856E-40
**ENSG00000158856**	**DMTN**	**521.1526385**	**1.931654325**	**4.84692E-39**
ENSG00000167994	RAB3IL1	330.4408154	2.15144047	2.03624E-37
**ENSG00000074410**	**CA12**	**6398.835475**	**1.970130223**	**8.69857E-37**
**ENSG00000003987**	**MTMR7**	**106.2107024**	**1.847210872**	**4.14666E-27**
**ENSG00000162891**	**IL20**	**268.9226145**	**5.82590076**	**1.20244E-24**
**ENSG00000136574**	**GATA4**	**89.24544754**	**6.003473563**	**1.41931E-23**
**ENSG00000188176**	**SMTNL2**	**96.65591498**	**3.258438859**	**2.11013E-15**
ENSG00000137628	DDX60	274.1966187	4.677465386	3.97364E-12
