# Supplementary material for: Genome engineering for estrogen receptor mutations reveals differential responses to anti-estrogens and new prognostic gene signatures for breast cancer
Source: Oncogene. 2022 Oct 5;41(44):4905–15. doi: 10.1038/s41388-022-02483-8 (PMC7613769; doi:10.1038/s41388-022-02483-8)
Supplement: Supplementary file 2 — Supplementary Figures [file 41388_2022_2483_MOESM2_ESM.pdf]

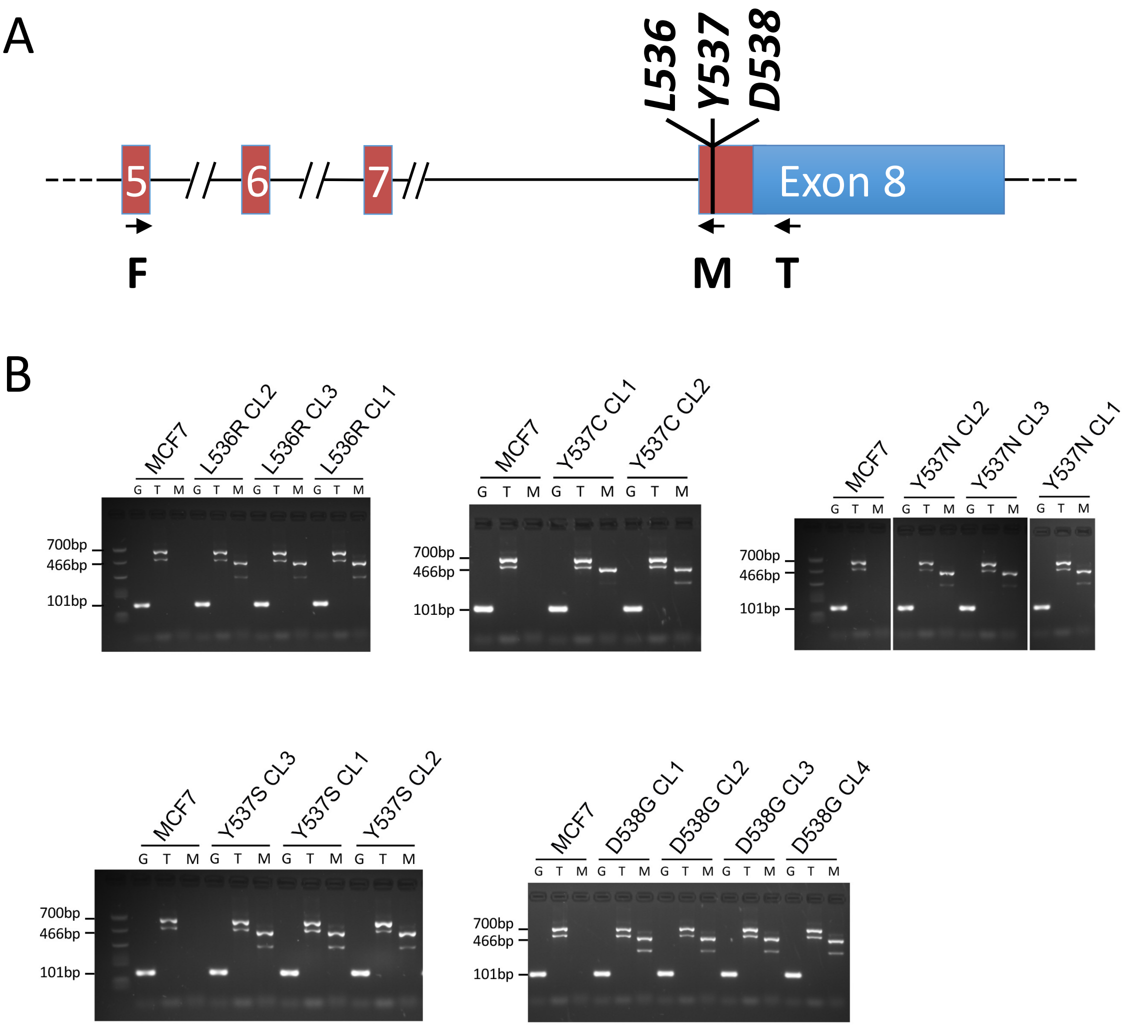

Supplementary Figure 1. RT-PCR demonstrates expression of mutant ER in CRISPR-Cas9 genome-edited clones

(**A**) Schematic showing the positions of RT-PCR primers within exons 5 and exon 8 (not drawn to scale). The same forward primer, F, was used with a primer in the 3' UTR of the ESR1 gene (primer T), to generate a 700 bp product, which would include WT and mutant ER transcripts. PCR using primer F with a reverse primer located within the region of mutation allows for specific amplification of mutant transcripts (product size = 466 bp). (**B**) RT-PCR of cDNA made from RNA prepared from mutant clones and from parental (MCF7) cells. Shown are agarose gel images. PCR using GAPDH primers are shown as a control for the cDNA (labelled as G). No product was observed for mutant-specific PCR in MCF7 cells. As described previously (Harrod *et al* 2017), the smaller PCR product for total and mutant-specific RT-PCR corresponds to an alternatively spliced ER mRNA lacking exon 7, that has previously been reported in MCF7 cells (see Herynk and Fuqua (2004) Estrogen receptor mutations in human disease. *Endocr Rev* 25: 869–898).

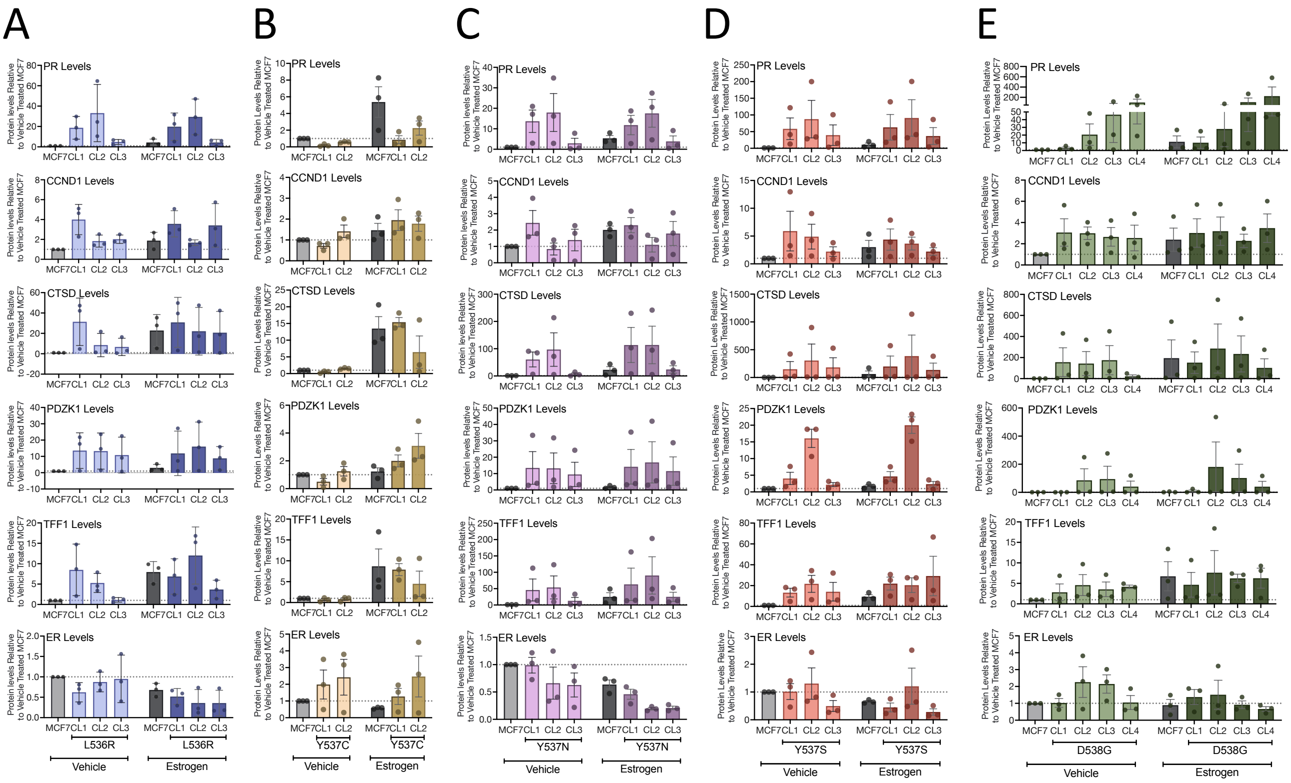

Supplementary Figure 2. Immunoblotting demonstrates elevated expression of ER-regulated genes in ESR1 mutant clones

(**A-E**) MCF7 cells and each of the L536R, Y537C, Y537N, Y537S and D538G mutant clones were cultured in estrogen-free culture medium for 72 hours. E2 (10 nM) or an equal volume of ethanol, was added to cultures for 16 hours. Immunoblotting was performed with protein lysates using antibodies for the indicated proteins. Quantification of immunoblotting of three independently generated sets of lysates was carried out with Image J (Schneider, C.A., Rasband, W.S., and Eliceiri, K.W. (2012). NIH Image to ImageJ: 25 years of image analysis. Nat Methods *9*, 671-675. 10.1038/nmeth.2089). The bar charts show levels of each protein relative to levels in vehicle-treated MCF7 cells, following normalisation against ß-actin. The actual immunoblotting images for one of the three replicates quantified here, are shown in Figure 1. Due to loading of protein lysates for multiple sets of mutant clones on the same gels, the same MCF7 loadings served as controls for different mutants in some experiments.

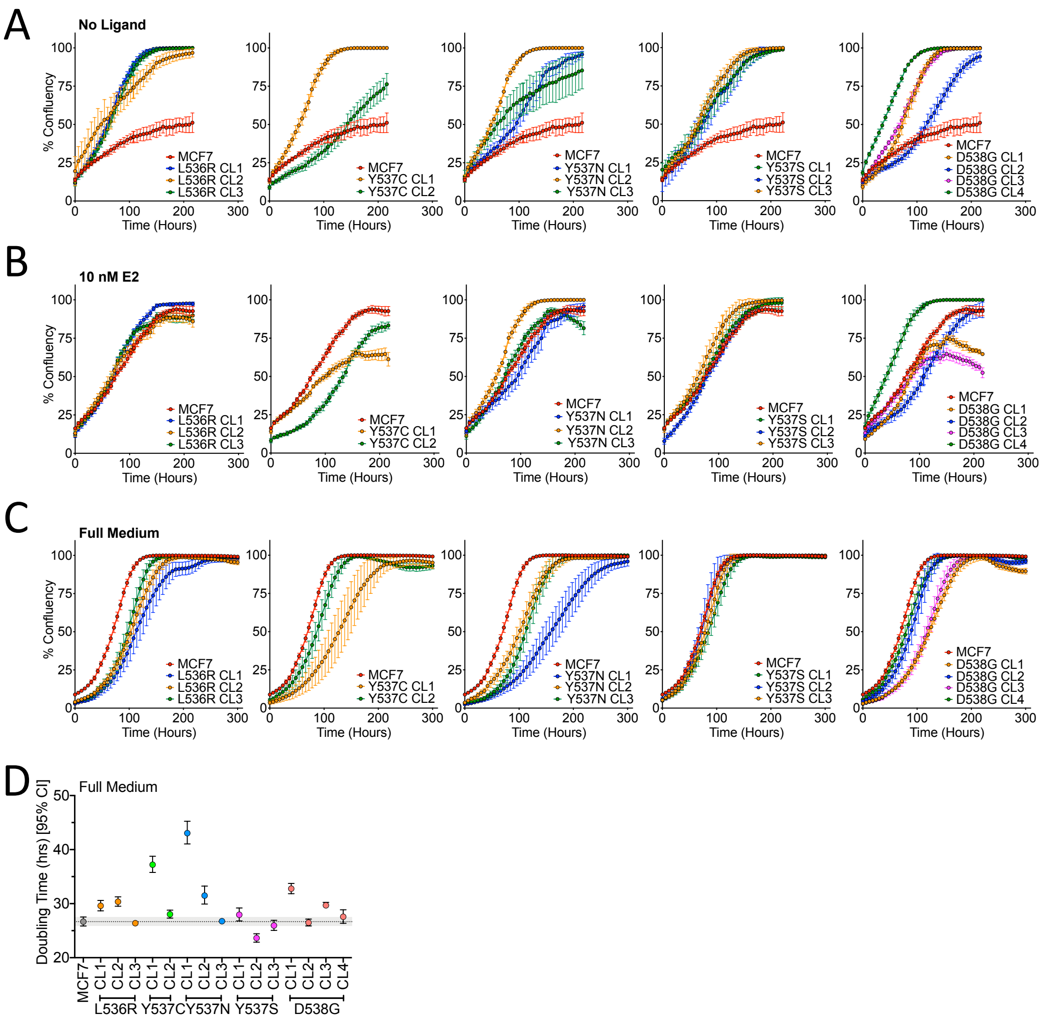

Supplementary Figure 3. MCF7 clones expressing all ER mutants show estrogen-independent growth

(**A-B**) 3000 cells were plated in estrogen-depleted medium in 96-well plates, in the presence of 10 nM E2 or the vehicle (DMSO, no ligand) and growth was imaged over 9 days, on an Incucyte Zoom (n = 5-6; error bars = 95% CI). (**C**) Cells, cultured in estrogen-replete (DMEM + 10% FCS) medium were treated as in A-B. All clones were assayed simultaneously, but clones for individual mutations are presented in separate graphs for ease of viewing. Thus, the same MCF7 data are plotted in each graph, for comparison. (**D**) Doubling times for each clone were determined using the exponential growth equation in Graphpad Prism (n = 6; error bars = 95% CI).

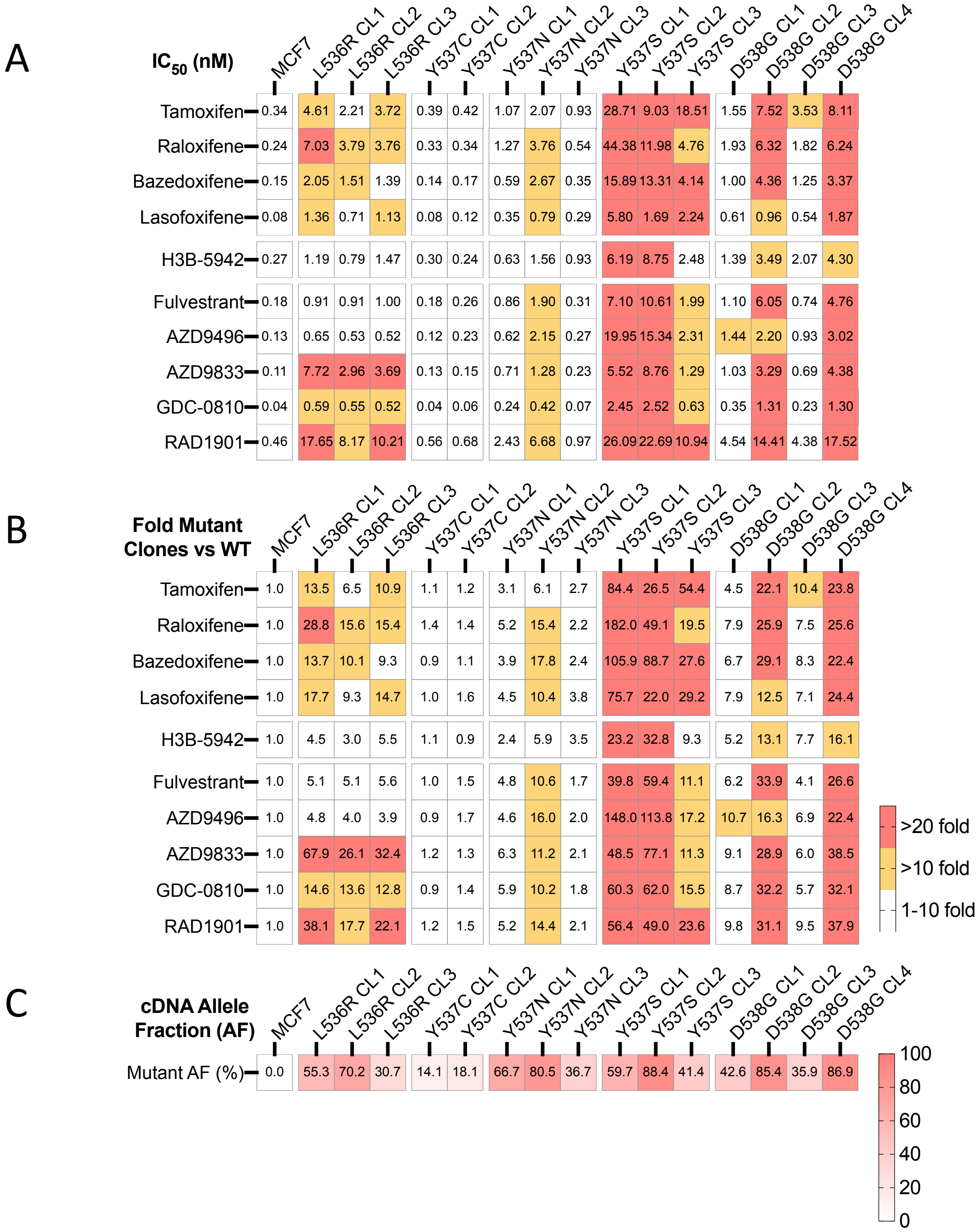

Supplementary Figure 4. Mutation-dependent resistance to anti-estrogens.

(**A**) IC_{50} values (nM) were established for each mutant clone, from 6-day growth in full medium (n=3 independent experiments). (**B**) The IC_{50} values in (A) are shown as fold relative to the IC_{50} values for MCF7-WT cells. (**A, B**) Values highlighted in orange show >2-fold difference from the IC_{50} value for MCF7 cells, with values in red identifying >10-fold difference in sensitivity to the drug. (**C**) Droplet digital PCR (ddPCR) of cDNA prepared from RNA for each mutant clone was used to determine level of expression of mutant ER in each clone. Mutant expression levels as percentage of total ER were calculated as mutant allele frequency (AF). These results are summarised as a heat map in Figure 2.

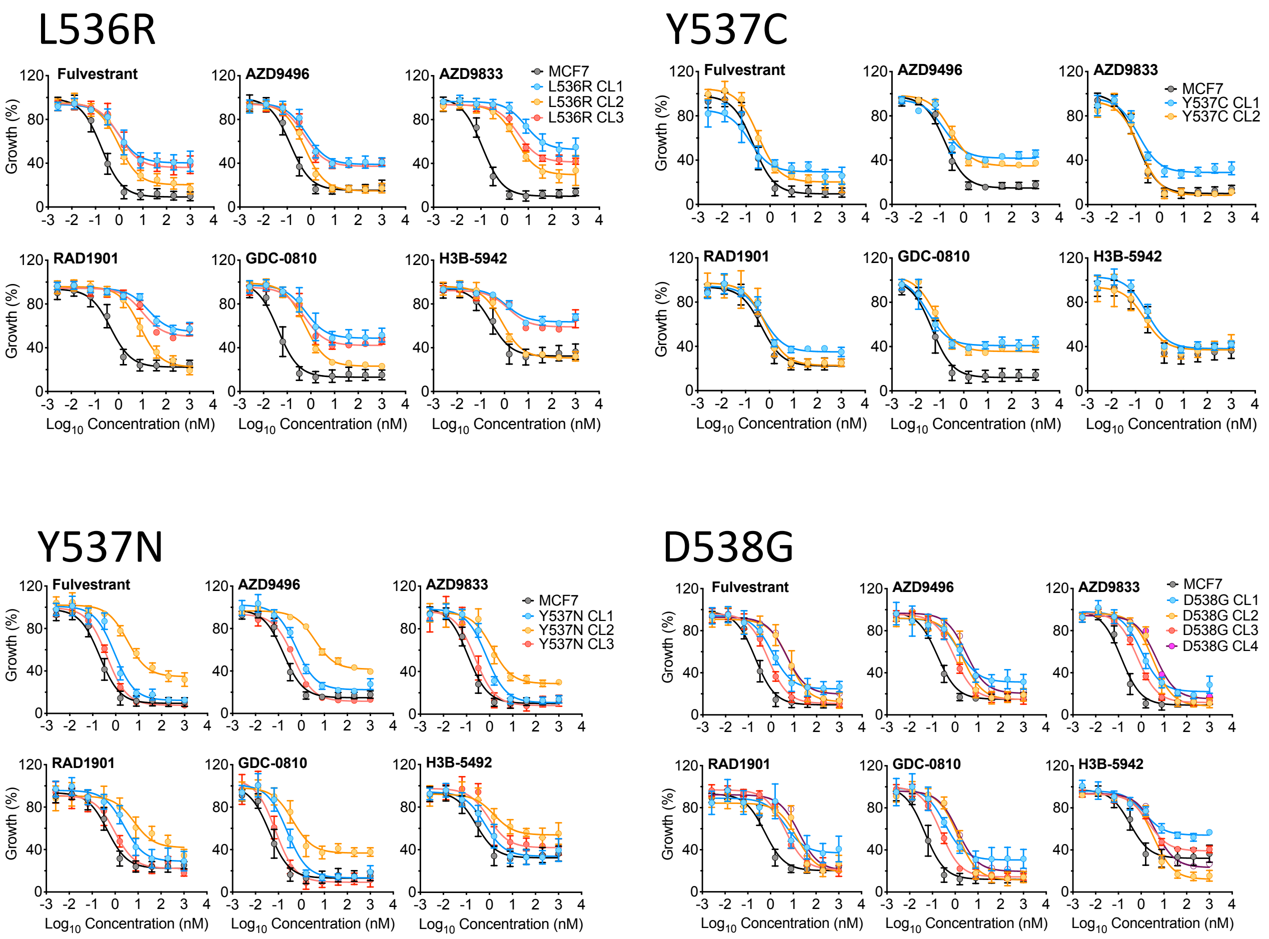

Supplementary Figure 5. ESR1 mutations promote resistance to anti-estrogens.

2,000 cells were plated in 96-well plates, in the presence of increasing concentrations of different anti-estrogens. Cell growth was determined after 6 days using the SRB assay. Growth is shown relative to to the vehicle control. The results for each mutant type and each anti-estrogen are shown separately, for ease of viewing. However, as all clones were assessed simultaneously for response to specific anti-estrogens, the same MCF7 data are depicted in the different graphs. Notwithstanding, the results are the means and standard deviation (error bars) for three independent experiments.

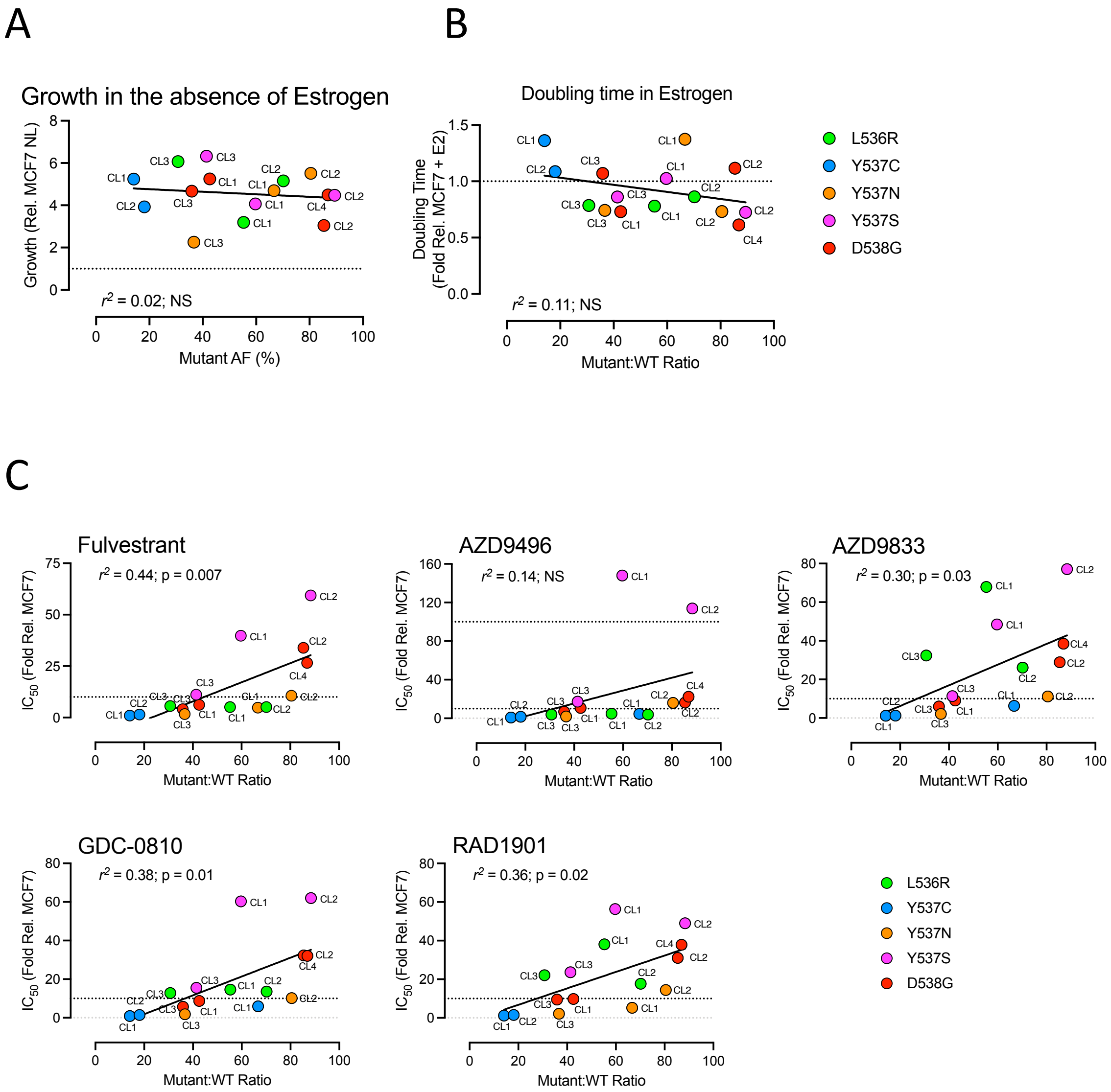

Supplementary Figure 6. Association between expression levels of ESR1 mutants and response to anti-estrogens

(**A**) Plotting fold difference in growth of ESR1 mutant clones is shown for estrogen-free (no-ligand (NL)) shows no association between levels of mutant expression and ligand-independent growth. (**B**) There is similarly no significant association between mutant expression levels and doubling time in estrogen-containing medium. (**C**) Plotting IC_{50} values relative to the IC50 values obtained for MCF7 cells, shows moderate positive correlation between mutant expression levels and resistance to SERDs. There was no significant correlation in the case of AZD9496. (**A-C**) Mutant expression levels are plotted as a percentage of total ER expression, determined from ddPCR of RNA prepared from each clone. Linear regression analysis was used to obtain goodness of fit (r^2) and *p*-values.

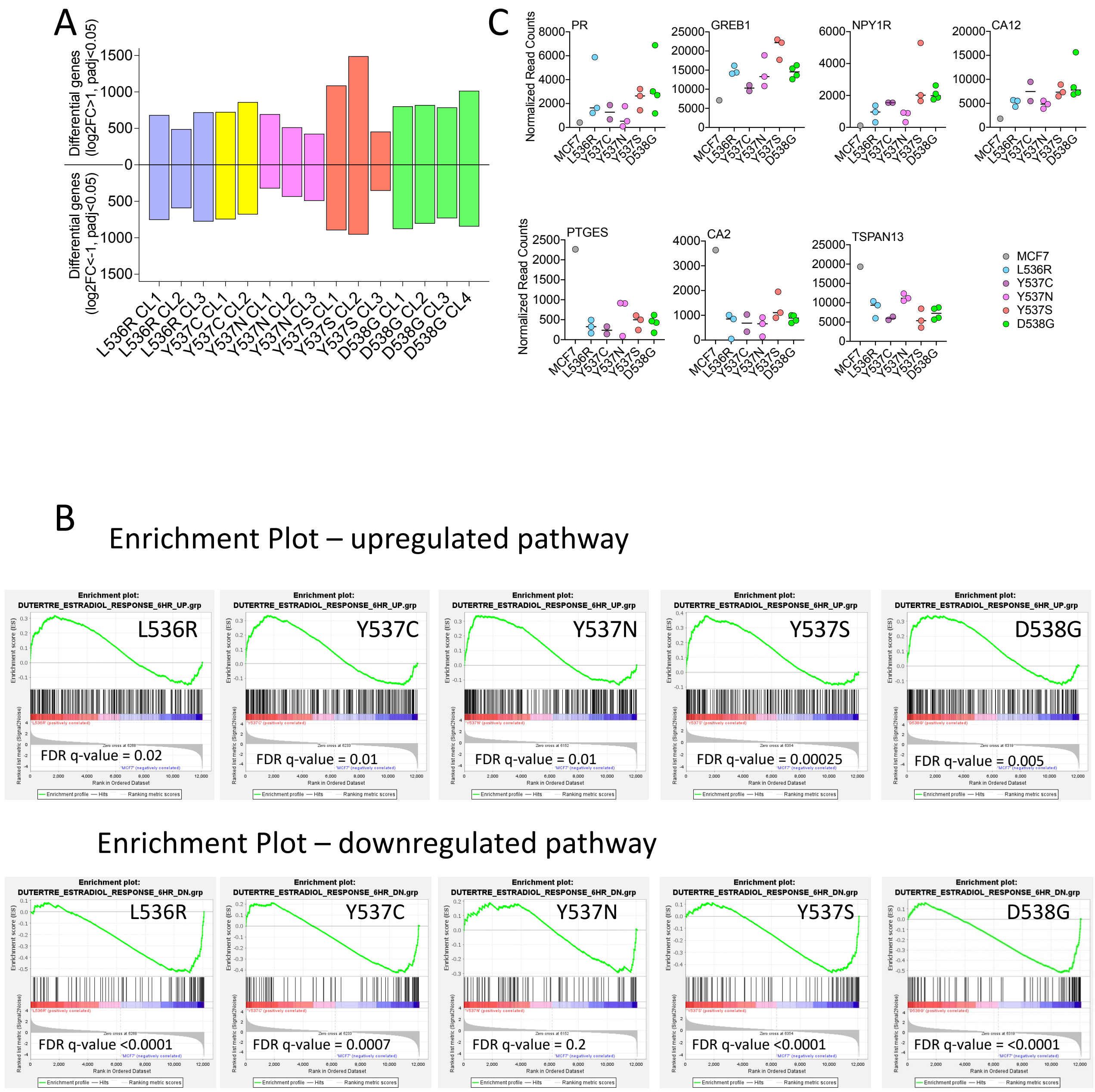

Supplementary Figure 7. Analysis of RNA-sequencing of ER mutant clones

(**A**) Differentially regulated genes, comparing each mutant clone with MCF7. Numbers of genes with higher (log2 FC>1), or lower log2 FC <-1) is depicted. (**B**) Enrichment plots from GSEA using curated gene sets involved in estrogen response. (**C**) Shown are the normalised read counts of exemplar estrogen-stimulated and estrogen-repressed genes. Each filled circle of the same colour shows an independent clone for the specific mutation.

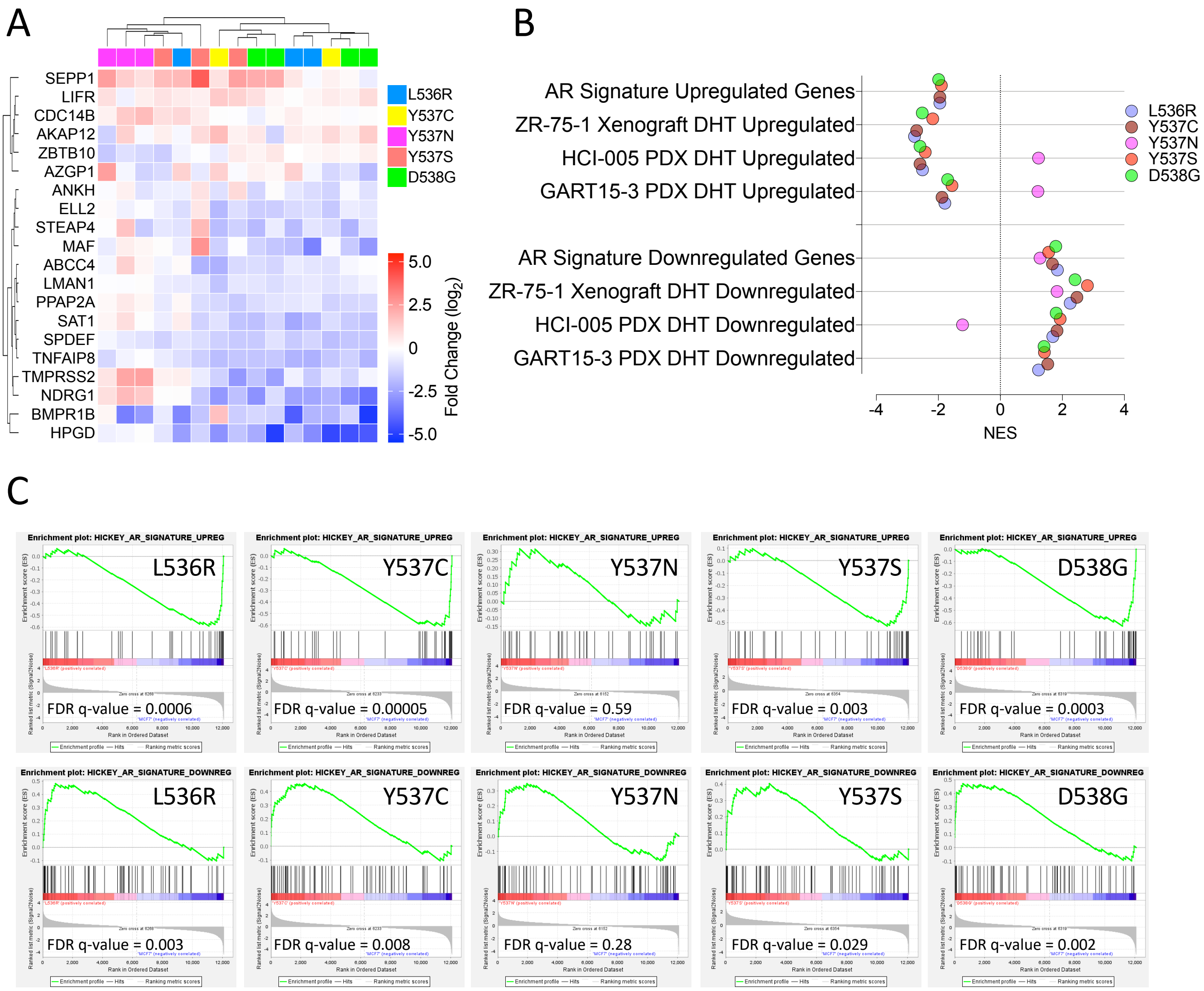

Supplementary Figure 8. Expression of Androgen-responsive genes is reduced in estrogen receptor mutant expressing breast cancer cells.

(**A**) Genes in the Hallmarks "Androgen Response" pathway was identified from GSEA analysis. The heatmap shows expression of these genes relative to expression in MCF7-WT. (**B**) Gene enrichment analysis was performed using androgen-responsive gene sets reported in NCBI GEO series GSE123770. Genes up-regulated by the androgen dihydrotestetosterone (DHT) were negatively enriched in the ESR1 mutant cell lines, whereas genes downregulated by DHT, were positively enriched. (**C**) The androgen-responsive gene sets in breast cancer, as described by Hickey et al (2021), were used within GSEA as custom gene sets for generation of GSEA enrichment plots.

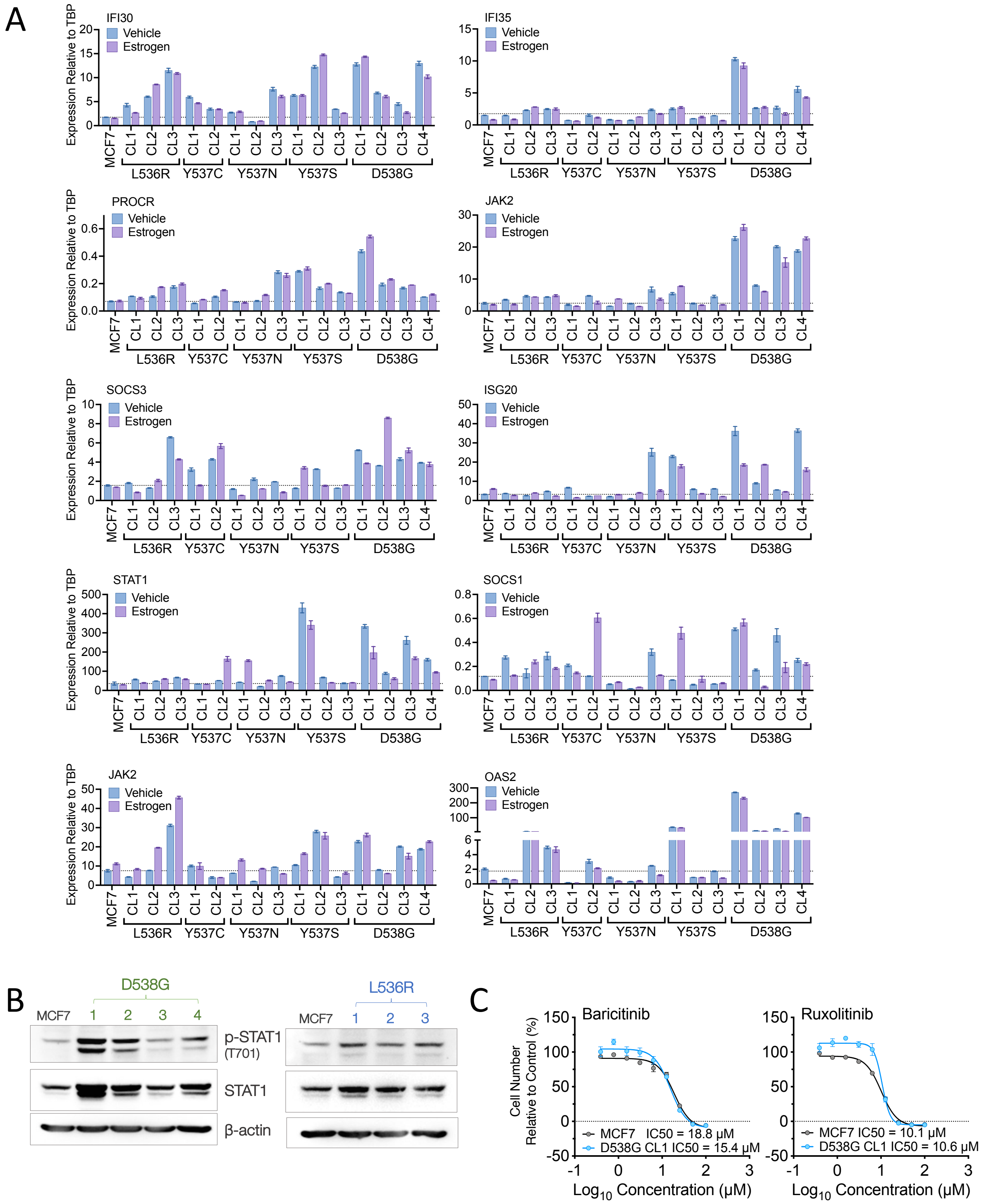

Supplementary Figure 9. Interferon-responsive gene expression is elevated in ESR1 mutant cells. (A) Cells cultured in the absence of estrogen were treated with 10 nM estrogen for 16 hours (n=3). Gene expression was normalised to expression of the housekeeping gene TBP. **(B)** Immunoblotting of cell lysates shows increased levels of STAT1 and phosphorylated STAT1 (p-T701) in ESR1 mutant clones. **(C)** 2,000 cells were plated in 96-well plates, in the presence of increasing concentrations of the JAK/STAT inhibitors, baricitinib or ruxolitinib. Cell growth was determined after 6 days using the SRB assay. Growth is shown relative to day 0.

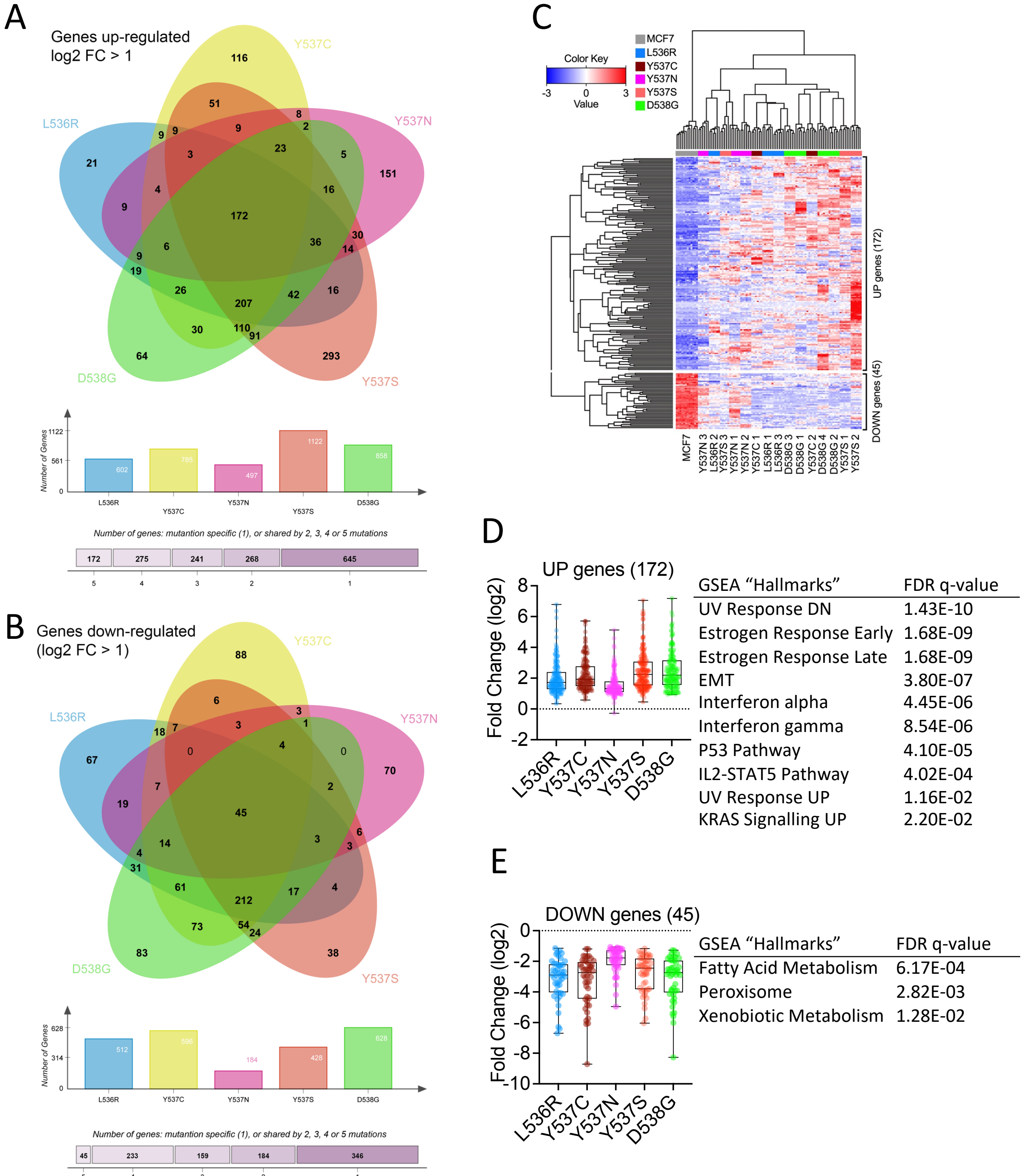

Supplementary Figure 10. The expression of a subset of genes is commonly altered in ESR1 mutant clones.

Differentially expressed genes (DEGs) were identified using DESeq2 for each ESR1 mutation relative to MCF7, with individual CRISPR-derived clones grouped together by mutation for this analysis. (**A**) DEGs, which have log2FC >1 and padj <0.05 in all ESR1 mutations are shown in the Venn diagram. (**B**) Down-regulated genes shared across ESR1 mutations (log2FC <-1, padj<0.05) are shown. (**C**) The heatmap shows unsupervised cluster analysis of regularised log transformed (rlog) read counts for each RNA-seq replicate for the up- and down-regulated genes in A-B. (**D-E**) The charts show average expression of each gene in all mutant clones for each ESR1 mutation (relative to MCF7 cells). Also shown are the top GSEA Hallmarks pathways for the up and down-regulated genes.

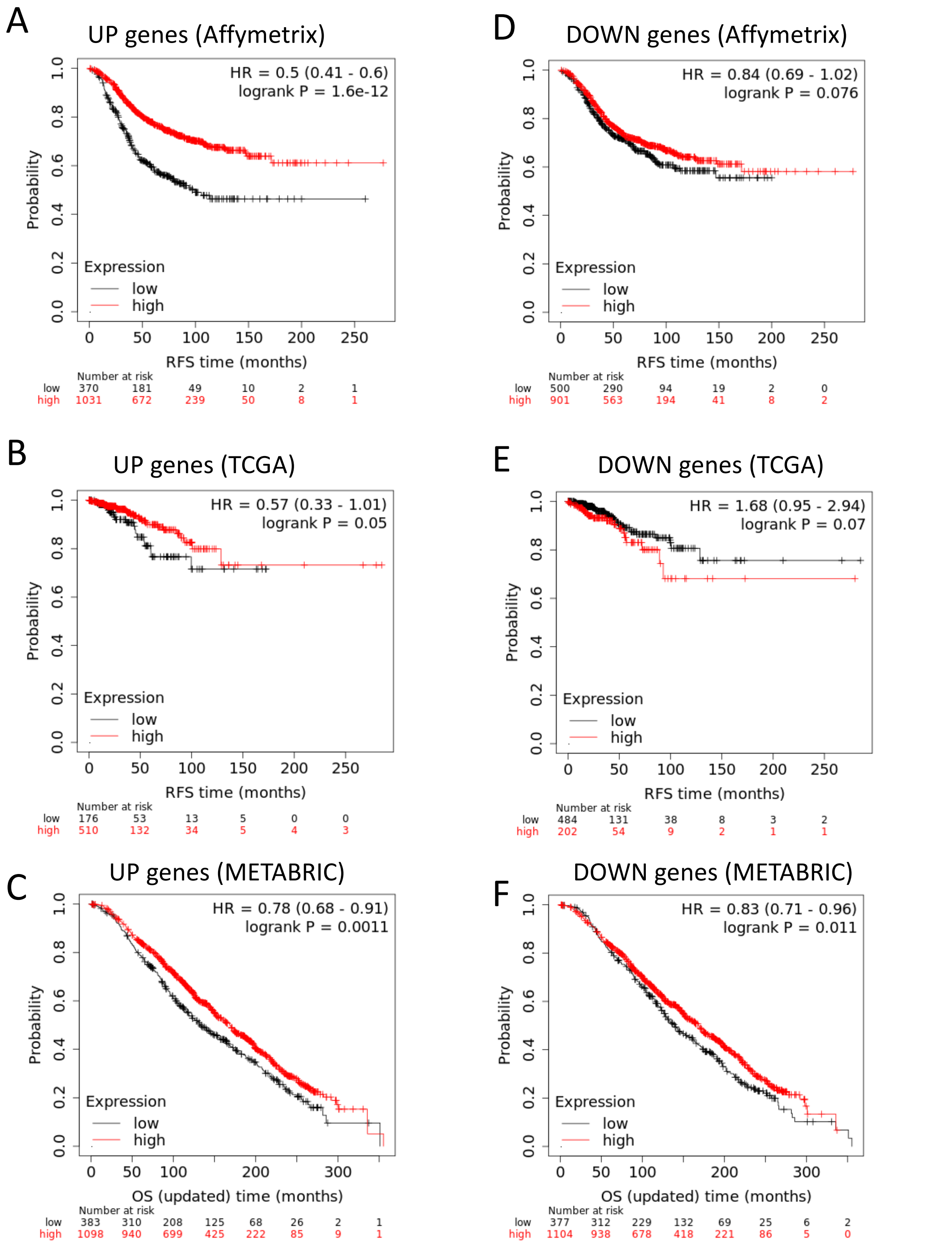

Supplementary Figure 11. Higher expression of genes up-regulated in ESR1 mutant breast cancer cells is associated with better outcome in ER+ breast cancer patients. (A) Kaplan-Meier plots of a database incorporating public Affymetrix microarray data series comprised of 1401 ER+ breast cancer patients. The 172 genes up-regulated in the combined clones for each ESR1 mutation relative to expression in MCF7 cells (log2FC<-1, p<0.05), were further refined by including only those genes with substantial expression in at least 42 RNA-seq samples having reads ≥50, thus reducing the number of genes used in the analysis to 143. (**B, C**) KM plot analysis of ER+ breast cancer samples in TCGA (n=686) and METABRIC (n=1481). *P*-values from the log rank test are shown. Hazard ratios, with 95% confidence intervals and log-rank p-values are also shown. RFS = relapse-free survival. (**D, E, F**) A similar analysis was undertaken for the 45 genes with reduced expression for all ESR1 mutations (log2FC<-1, p<0.05).

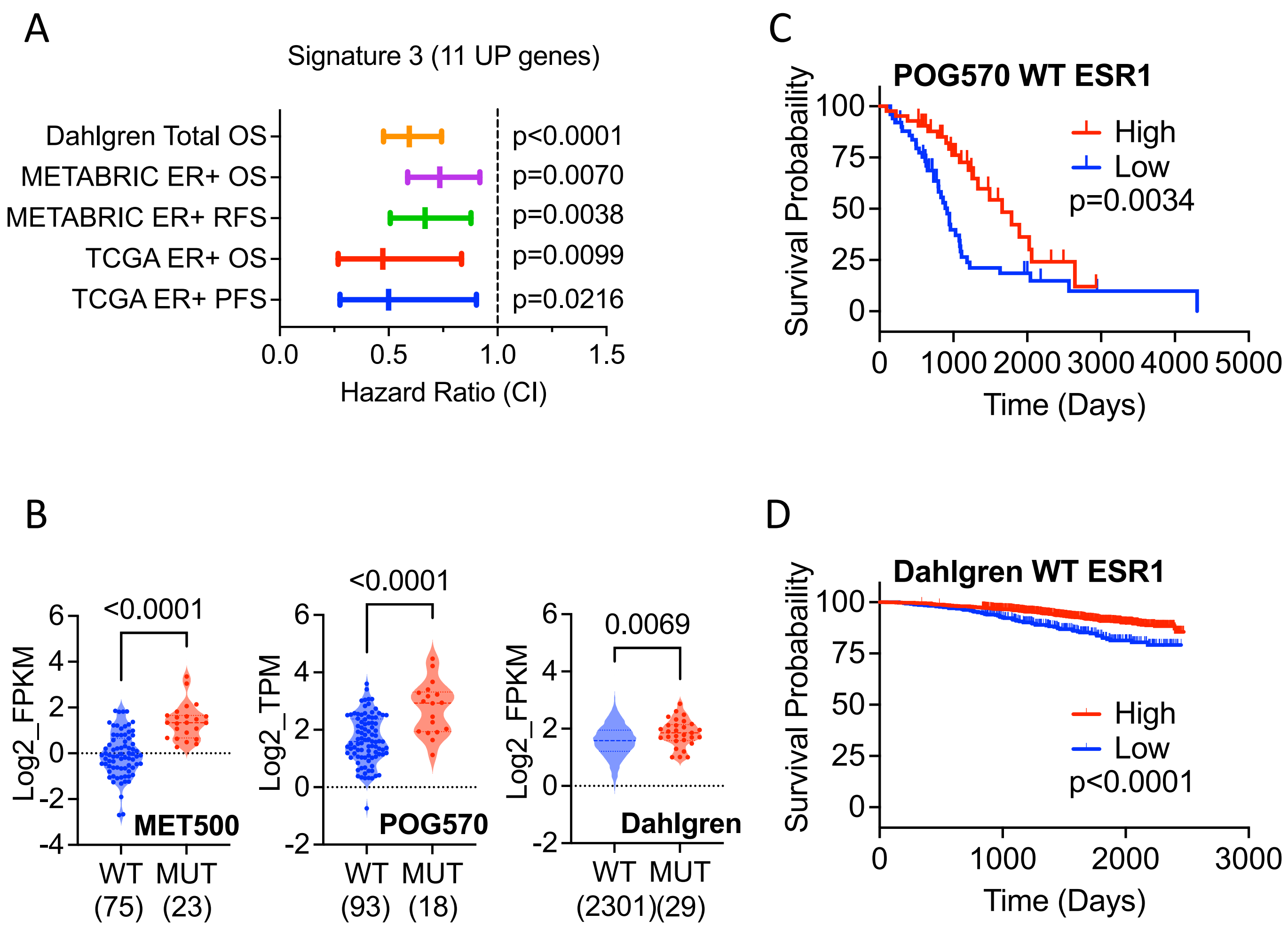

Supplementary Figure 12. Genes upregulated by ESR1 mutant expression are associated with ER+ breast cancer patient outcome.
(A) 15 genes were significantly up-regulated in all clones for each ESR1 mutation. Four genes were excluded as non-coding genes and low read counts in the majority of replicates. The remaining 11 genes were used for univariate Cox proportional hazards analysis in the survminer R package. TCGA pan cancer atlas downloaded from cbioportal (5th Jan 2022) – expression: RNA_Seq_v2_mRNA_median_all_sample_Zscores), patient number n=794. METABRIC: downloaded from cbioportal (~20 July 2021) mRNA_median_Zscores, patient number n=1444. Dahlgren: Log2_FPKM ER+ patient data obtained from Dahlgren et al 2021, n=2720). (**B**) Expression of the 11-gene list is elevated in ESR1 mutant metastatic (POG570, MET500) and primary (Dahlgren) breast cancer. The number of ER+ patients with WT and mutant ESR1 are shown in parentheses. The Mann-Whitney test was used for significance determination. (**C-D**) KM plots of survival for ER+ breast cancer patients with WT ESR1. The hazard ratios (HR), together with 95% confidence intervals (in brackets) and *p* value from the log rank test is shown in each panel. KM plots were generated in Survminer, using the six gene signature.
