# Supplementary material for: Genome engineering for estrogen receptor mutations reveals differential responses to anti-estrogens and new prognostic gene signatures for breast cancer
Source: Oncogene. 2022 Oct 5;41(44):4905–15. doi: 10.1038/s41388-022-02483-8 (PMC7613769; doi:10.1038/s41388-022-02483-8)
Supplement: Supplementary file 3 — Supplementary Information [file 41388_2022_2483_MOESM3_ESM.docx]

**Reagents**

| Oligonucleotides for site-directed mutagenesis of ESR1 template for genome editing | |  |
| --- | --- | --- |
| Mutation | **Sequence 5’ -> 3’** | |
| L536R | CCAGCAGCAGGTCATACCGGGGCACCACGTTC | |
| Y537C | GAACGTGGTGCCCCTGTGTGACCTGCT | |
| Y537N | GAACGTGGTGCCCCTGAATGACCTGCTGCTGG | |
| Y537S | GAACGTGGTGCCCCTGTCTGACCTGCTGCTG | |
| D538G | CAGCATCTCCAGCAGCAGACCGTACAGGGGCACCACGTTCT | |
| CRISPR19 targeting site mutations | TGCTGGAGATGCTGGACGCCCATAGACTTCACGCGCCCACTAGC | |
| CRISPR92/93 PAM site mutations | GTCTTTGGAGTTCCTCTTCCTTGGGGTTCTAGGGATTTCAGCACTC | |

| CRISPR Sequences | |  |
| --- | --- | --- |
| Mutation | **Sequence 5’ -> 3’** | |
| CRISPR 19, ESR1 exon 8 chr6: 152,419,954 - 152,419,976 (GRCh37/hg19) | GGCTAGTGGGCGCATGTAGG | |
| CRISPR 92, ESR1 intron 7 chr6: 152,419-816 - 152,419,795 (GRCh37/hg 19) | GGAGTGCTGAAATCCCTAGA | |
| CRISPR 93, ESR1 intron 7 chr6: 152,419,815 - 152,419,794 (GRCh37/hg 19) | GAGTGCTGAAATCCCTAGAA | |

| CRISPR sgRNAs used in the generation of ESR1 mutant clones | |  |
| --- | --- | --- |
| Mutant Clone | **CRISPR sgRNA used** | |
| L536R CL1, CL2, Cl3 | CRISPR4834192 | |
| Y537C CL1, Cl2 | CRISPR4834192 | |
| Y537N CL1, CL2, CL3 | CRISPR4834192 | |
| Y537S CL1 | CRISPR4834192 | |
| Y537S CL2 | CRISPR4834193 | |
| Y537S CL3 | CRISPR19 | |
| D538G CL1, CL2, CL3, CL4 | CRISPR4834192 | |

| Droplet digital PCR (ddPCR) | | | | |
| --- | --- | --- | --- | --- |
|  | **Probe 5’ -> 3’** | **Tm without quencher** | **Length (bp)** | **Reporter/Quencher** |
| WT | TCTCCAGCAGCAGGTCATAGA | 65 | 21 | VIC/MGB |
| L536R | CTCCAGCAGCAGGTCATACC | 64.8 | 20 | FAM/MGB |
| Y537C | CAGCAGCAGGTCACACAGG | 66.1 | 19 | FAM/MGB |
| Y537N | CCAGCAGCAGGTCATTCAG | 64.8 | 19 | FAM/MGB |
| Y537S | CCAGCAGCAGGTCAGACA | 64.2 | 18 | FAM/MGB |
| D538G | CCAGCAGCAGACCGTACA | 63.9 | 18 | FAM/MGB |
|  | **Common ddPCR primers**  **5’ -> 3’** | **Tm without quencher** | **Length (bp)** |  |
| Forward | TAACAAAGGCATGGAGCATCTG | 65 | 21 |  |
| Reverse | AGGAATGCGATGAAGTAGAGCC | 63.9 | 18 |  |

| Primers Used For RT-qPCR | |  |
| --- | --- | --- |
| Gene | **Sequence 5’ -> 3’** | |
| TBP | Forward: GTTTGCCAAGAAGAAAGTGAAC  Reverse: GGGTCAGTCCAGTGCCAT | |
| ESR1 | Forward: AAGAAAGAACAACATCAGCAGTAAA  Reverse: TCTTTGGATGACCTAGCCTCTC | |
| PGR | Forward: GAGTCACCTTGCCCCTTCA  Reverse: GCCTTTTATGCTTGCCTGTAAA | |
| GREB1 | Forward: ATAAGAGAATCCACAATAGAGACCTT  Reverse: CTCTCATCAGTTTCAGTGCCATTT | |
| TFF1 | Forward: CCCCCGTGAAAGACAGAATT  Reverse: ACGTCGATGGTATTAGGATAGAAG | |
| PDZK1 | Forward: TTCCTGCGAATTGAGAAGGAC  Reverse: TCCACCCGTGTTTTCACTGC | |
| EGR3 | Forward: GACATCGGTCTGACCAACGAG  Reverse: GGCGAACTTTCCCAAGTAGGT | |

| Compounds | |  |  |
| --- | --- | --- | --- |
| Name | **Cat. No.** | | **Supplier** |
| 17ß-estradiol (E2) | E8875 | | sigma-aldrich merck, dorset, uk |
| 4-hydroxytamoxifen (OHT) | H7904 | | sigma-aldrich merck, dorset, uk |
| Raloxifene | 2280 | | Tocris Bioscience, Bio-Techne, Abingdon, UK |
| Bazedoxifene | 5263 | | Tocris Bioscience, Bio-Techne, Abingdon, UK |
| Lasofoxifene | C5637 | | APExBIO, Boston, USA |
| AZD9496 |  | | Astra Zeneca, Cambridge, UK |
| AZD9833 |  | | Astra Zeneca, Cambridge, UK |
| GDC-0810 |  | | Genentech Inc, San Francisco, USA |
| RAD1901 | HY-19822A | | MedChemExpress, Insight Biotechnology Ltd, UK |
| H3B-5942 | HY-112611 | | MedChemExpress, Insight Biotechnology Ltd, UK |

| Primary and Secondary Antibodies | | | | |
| --- | --- | --- | --- | --- |
| Antibody | **Species** | **Supplier** | **Cat. No.** | **Dilution** |
| ß-actin | Mouse | Abcam (Cambridge, UK) | ab6276 | 1:100,000 |
| ER | Mouse | Novocastra (Newcastle upon Tyne, UK) | NCL-L-ER-6F11 | 1:1000 |
| CCND1 | Rabbit | Abcam | ab16663 | 1:200 |
| CTSD | Mouse | Abcam | ab6313 | 1:1000 |
| PDZK1 | Rabbit | ProteinTech (Manchester, UK) | 10507-2-AP | 1:500 |
| TFF1 | Rabbit | Santa Cruz (California, USA) | sc-28925 | 1:200 |
| PR | Rabbit | Cell Signalling Technology | 8757 | 1:500 |
| Anti-mouse igG-HRP | Goat polyclonal | Dako (Agilent) | P0447 | 1:2000 |
| Anti-rabbit IgG-HRP | Goat polyclonal | Dako | P0448 | 1:2000 |
| Anti-rat IgG-HRP | Rabbit polyclonal | Dako | P0450 | 1:2000 |
